# Assisted clustering of gene expression data using regulatory data from partially overlapping sets of individuals

**DOI:** 10.1186/s12864-022-09026-1

**Published:** 2022-12-10

**Authors:** Wenqing Jiang, Roby Joehanes, Daniel Levy, George T O’Connor, Josée Dupuis

**Affiliations:** 1 Department of Biostatistics, Boston University School of Public Health, MA Boston, USA; 2 National Heart, Lung, and Blood Institute’s Framingham Heart Study, MA Framingham, USA; 3 The Population Sciences Branch, National Heart, Lung, and Blood Institute, National Institutes of Health, MD Bethesda, USA; 4 Department of Medicine, Pulmonary Center, Boston University, MA Boston, USA

**Keywords:** Multi-omics data integration, Gene expression, Clustering, DNA methylation, Genotype, Framingham Heart Study

## Abstract

**Background:**

As omics measurements profiled on different molecular layers are interconnected, integrative approaches that incorporate the regulatory effect from multi-level omics data are needed. When the multi-level omics data are from the same individuals, gene expression (GE) clusters can be identified using information from regulators like genetic variants and DNA methylation. When the multi-level omics data are from different individuals, the choice of integration approaches is limited.

**Methods:**

We developed an approach to improve GE clustering from microarray data by integrating regulatory data from different but partially overlapping sets of individuals. We achieve this through (1) decomposing gene expression into the regulated component and the other component that is not regulated by measured factors, (2) optimizing the clustering goodness-of-fit objective function. We do not require the availability of different omics measurements on all individuals. A certain amount of individual overlap between GE data and the regulatory data is adequate for modeling the regulation, thus improving GE clustering.

**Results:**

A simulation study shows that the performance of the proposed approach depends on the strength of the GE-regulator relationship, degree of missingness, data dimensionality, sample size, and the number of clusters. Across the various simulation settings, the proposed method shows competitive performance in terms of accuracy compared to the alternative K-means clustering method, especially when the clustering structure is due mostly to the regulated component, rather than the unregulated component. We further validate the approach with an application to 8,902 Framingham Heart Study participants with data on up to 17,873 genes and regulation information of DNA methylation and genotype from different but partially overlapping sets of participants. We identify clustering structures of genes associated with pulmonary function while incorporating the predicted regulation effect from the measured regulators. We further investigate the over-representation of these GE clusters in pathways of other diseases that may be related to lung function and respiratory health.

**Conclusion:**

We propose a novel approach for clustering GE with the assistance of regulatory data that allowed for different but partially overlapping sets of individuals to be included in different omics data.

**Supplementary Information:**

The online version contains supplementary material available at 10.1186/s12864-022-09026-1.

## Background

DNA microarray technology has made it possible to study gene expression that characterizes important biological processes across collections of different or related individuals. Elucidating the co-expression structure in the GE and discovering genes that have similar behavior under some conditions but behave independently under other conditions offer a tremendous opportunity for an enhanced understanding of functional genomics. A great deal of research is being carried out on the algorithms for clustering GE data [1]. Many of the clustering algorithms that are popular today are distance based [2]. The most widely used clustering algorithms for gene expression data include hierarchical clustering (HC) [3], K-means [4], and self-organizing maps (SOMs) [5]. HC algorithm is one of the earliest clustering algorithms for clustering GE data. However, it has been reported that HC can cause points to be clustered largely based on local decisions – the iterative mergences are determined locally by the pairwise distances instead of a global criterion [6]. HC can be highly vulnerable if genes are scattered [7]. K-means clustering is based on a random selection of initial seed point of preferred clusters. It is quite computationally efficient but can be sensitive to outliers [6]. SOMs is a model-based clustering algorithm that maps high-dimensional data into 2D or 3D space [8]. It is widely used for GE clustering; however the fact that SOMs attempt to merge different patterns into a cluster can make it ineffective and produce unstable solutions [9]. These algorithms are quite simple and visually appealing, but their performances could all be sensitive to noise [10, 11].

Recent high-throughput technologies have generated a large amount of omics data. As omics measurements profiled on different molecular layers are interconnected, integrative approaches that incorporate the regulatory effect from multi-level omics data are needed. Borrowing strength across multi-level omics data makes integration more comprehensive than single-level analysis. The ideal situation would be when the multi-level omics data, for example, gene expressions (GE) and their regulators (copy number variation CNV, microRNA, methylation, etc.), are measured on the same individuals, making it possible to incorporate information across different molecular layers. Under this situation, there are several options to perform data integration, including Assisted Normalized Cut (ANCut), a clustering approach of GE with the assistance of information from regulators, developed by Hidalgo et al. [12]. However, a more realistic situation would be that the multi-omic data are not measured on the same individuals, for example, when we have access to gene expression profiles from one set of individuals and DNA methylation profiles from another set of individuals. There may be some overlap between the two sets of individuals but the individuals included in the two datasets are not completely overlapping. Under this scenario, the availability of approaches for integration of GE and methylation is limited. In this paper, we develop a new method to cluster gene expression data with integration from other omic data types, when data come from different but partially overlapping sets of individuals. Our method borrows strengths from the previously developed ANCut approach.

Our ultimate goal is to better understand the biological mechanisms that lead to the development of a particular disease. The biological mechanisms may be described by a series of steps, and at each step, the activity of an entity alters the state of another entity [13]. Genes with similar expression patterns under various conditions may imply co-regulation or relation in functional pathways [14]. To investigate how genes interconnect and function with upstream regulators, we propose to improve GE clustering by integrating regulatory data from different but partially overlapping sets of individuals. The rationale is to decompose gene expression into the regulated component and the other component that is not regulated by measured factors. Gene expression is typically measured with error, and by using the regulated component of gene expression, the clusters may be better defined – as long as the clustering structure of the genes is due mostly to the regulated component, rather than the non-regulated component. This decomposition structure has been extensively used in many previously developed integrative genome analysis approaches, including ANCut [12], PrediXcan [15], and iBAG [16]. ANCut, proposed by Hidalgo et al., uses a two-stage framework to conduct integrative clustering analysis on gene expression [12]. First, important GE-regulator relationship is identified through elastic net where the correlations among regulators (such as CNVs) can be properly accounted for [12]. Then, the ANCut measure incorporating weight matrices corresponding to both original and regulated GEs is adopted to cluster GE [12]. PrediXcan estimates the component of gene expression determined by an individual’s genetic profile and correlates the “imputed” gene expression with the phenotype under investigation to identify genes involved in the etiology of the phenotype [15]. PrediXcan application to a GWAS dataset consists of “imputing” the transcriptome using the weights derived from reference transcriptome datasets and correlating the genetically regulated GE component with the phenotype of interest using regression methods or non-parametric approaches. iBAG, propsed by Wang et al., decomposes GE into two components at the level of mechanistic model, one component directly regulated by its regulators and the other component influenced by other mechanisms [16]. The association between patients’ survival is modeled as a function of the two components of GE [16]. Both ANCut and iBAG require complete overlap of individuals between omics datasets. PrediXcan requires external reference data for application, and the overlap of individuals between reference omics datasets needs to be complete as well. In our analysis, we do not need any reference data or require exactly the same set of individuals across different omic measurements. Instead, a certain fraction of overlapping individuals between GE data and the regulatory data is adequate for modeling the regulation, thus improving GE clustering.

## Methods

### Modeling the GE-methylation regulation

In the next sections, we use methylation data as an example regulator to demonstrate the proposed approach. However, the method is generalizable to incorporate other types of regulators of interest that can be quantified (for example, regulatory genetic variants, lncRNA, microRNA, and copy number variation). Consider a dataset with $n={n}_{1}+{n}_{2}+{n}_{3}$ independent individuals, indexed by $i$. For individuals$i=1,\dots ,\left({n}_{1}+{n}_{2}\right)$, we assume that measurements are available on $p$ GEs, denoted as ${\varvec{Y}}_{\varvec{i}}=\left({Y}_{i1},{Y}_{i2},\dots ,{Y}_{ip}\right)$, $i=1,\dots ,\left({n}_{1}+{n}_{2}\right)$. In addition, for individuals $i=\left({n}_{1}+1\right),\dots ,\left({n}_{1}+{n}_{2}+{n}_{3}\right)$, we assume that measurements are available on $q$ methylation sites, denoted as ${\varvec{X}}_{\varvec{i}}=\left({X}_{i1},{X}_{i2},\dots ,{X}_{iq}\right), i=\left({n}_{1}+1\right),\dots ,\left({n}_{1}+{n}_{2}+{n}_{3}\right)$. For individuals $i=\left({n}_{1}+1\right),\dots ,({n}_{1}+{n}_{2})$, both the GE measurements and methylation measurements are available, which enables modeling of the GE-methylation regulation based on these overlapping individuals. Specifically, consider ${Y}_{ij}={\varvec{X}}_{\varvec{i}}{\varvec{\beta }}_{\varvec{j}}+\in_{ij}, i=\left({n}_{1}+1\right),\dots ,\left({n}_{1}+{n}_{2}\right), j=1,\dots ,p$

where ${\varvec{\beta }}_{\varvec{j}}$ is the vector of the unknown regression coefficients of gene $j$ with dimension $q\times 1$, and $\in_{ij}$ is the random error, or in the matrix form

$$Y=X\beta+\epsilon$$

where $\varvec{\beta }=\left({\varvec{\beta }}_{1},\dots ,{\varvec{\beta }}_{\varvec{p}}\right)$ is the matrix of unknown regression coefficients with dimension $q\times p$, and $\in=\left(\in_{ij}\right)$ is a matrix of random errors.

We model the GE-methylation regulation relationship for one gene at a time,

$$Y_j=X\beta_j+\in_j,\;j=1,\dots,p$$

where $\in_j=\left(\in_{\left(n_1+1\right)j},\dots,\;\in_{\left(n_1+n_2\right)j}\right)$ is the vector of random errors.

To estimate $\varvec{\beta }=\left({\varvec{\beta }}_{1},\dots ,{\varvec{\beta }}_{\varvec{p}}\right)$, we consider the following penalized estimate for each gene $j$,

1 $${\widehat\beta}_j=\underset{\beta_j}{\text{argmin}}\left\{\left\|Y_j^O-X^O\beta_j\right\|_2^2+\lambda_j\left(\left(1-\alpha_j\right)\left\|\beta_j\right\|_2^2+\alpha_j{\left\|\beta_j\right\|}_1\right)\right\},$$

where $Y_j^O$ is the vector consisting of $Y_{ij}$’s of the overlapping individuals, $i=\left(n_1+1\right),\dots,\left(n_1+n_2\right),$ with dimension ${n}_{2}\times 1$; ${\varvec{X}}^{\varvec{O}}$ is the matrix consisting of ${\varvec{X}}_{\varvec{i}}$’s of the overlapping individuals, $i=\left({n}_{1}+1\right),\dots ,\left({n}_{1}+{n}_{2}\right),$ with dimension ${n}_{2}\times q$ respectively; and ${\lambda }_{j}>0$ and $0\le {\alpha }_{j}\le 1$ are data-dependent tuning parameters of gene $j$, as proposed by Hidalgo et al. [12].

We assume that only a subset of the individuals has both GE and methylation data, but our approach is equivalent to that of Hildago et al. when both omic data are available from all individuals [12]. In our approach, the regulation model is constructed based only on the overlapping individuals instead of the full data, adding flexibility to the approach. Moreover, we propose methods to estimate the optimal number of clusters, and these estimation methods can be applied to both our novel method and the original approach assume complete overlap between omics datasets.

**Fig. 1:**
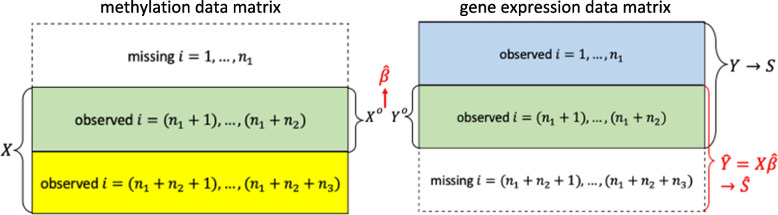
Visualization of GE-methylation regulation model data structure and framework for the proposed assisted clustering method

The data structure is visually described in Fig. 1.

A penalization approach is adopted to accommodate the high data dimensionality and for variable selection purposes: for a specific gene, its expression level is determined by only a few methylation sites. To address the potential correlation among methylation at different sites, the elastic net penalty is adopted. In data analysis, this estimation is effectively realized using the R package $glmnet$. The two tuning parameters, $\lambda$ and $\alpha$, are selected using V-fold cross-validation (V = 5 in our simulation study and data analysis). The expression level of a gene is usually regulated by methylation located near the transcription start site since distant methylation sites would be less likely to regulate a given gene. For this reason, we believe genes have different methylation regulators. Thus, we model one gene at a time to allow different sets of methylation predictors for each gene.

With the estimate $\widehat{\varvec{\beta }}$, we denote the predicted GE values as $\widehat{\varvec{Y}}=\varvec{X}\widehat{\varvec{\beta }}$, where $\varvec{X}$ is the matrix consisting of ${X}_{i}$’s of all the individuals with available methylation measurements, $i=\left({n}_{1}+1\right),\dots ,\left({n}_{1}+{n}_{2}+{n}_{3}\right),$ with dimension $\left({n}_{2}+{n}_{3}\right)\times q$. This captures the component of GEs regulated by the methylation sites included in $X$ for individuals $i=\left({n}_{1}+1\right),\dots ,\left({n}_{1}+{n}_{2}+{n}_{3}\right)$.

### Assisted clustering with partially overlapping sets of individuals

Consider the similarity matrix $\varvec{S}={\left({s}_{jl}\right)}_{p\times p}$, where the non-negative element ${s}_{jl}$ measures the similarity between genes $j$ and $l$. We define ${s}_{jl}$ to be equal to the inverse of their Euclidean distance between the original GE measurements ($\varvec{Y}$) for genes $j$ and $l$. Further, we define $\widehat{\varvec{S}}$, which is obtained in a similar way as $\varvec{S}$ but using $\widehat{\varvec{Y}}=\varvec{X}\widehat{\varvec{\beta }}$, the predicted regulated component of GEs given $\varvec{X}$. Note that $\varvec{Y}$ and $\widehat{\varvec{Y}}$ are available on a different set of individuals, because the GE data and methylation data come from different sets of individuals.

Denote ${A}_{1},\dots ,{A}_{K}$ as a partition of $\left\{1,\dots ,p\right\}$ which leads to $K$ disjoint clusters of genes, assuming $K$ is known. For ${A}_{k}$, denote ${A}_{k}^{c}$ as its complement. We define the ANCut.overlap measure of between-cluster similarity over the within-cluster similarity ratio for each cluster,

2 $$ANCut.overlap\left({A}_{k}\right)=\frac{cut\left({A}_{k},{A}_{k}^{c};\varvec{S}\right)}{cutvol\left({A}_{k};\widehat{\varvec{S}}\right)},$$

where

3 $$cut\left({A}_{k},{A}_{k}^{c};\varvec{S}\right)=\sum\limits_ {j\in {A}_{k},l\in {A}_{k}^{c}}{s}_{jl},$$

and

4 $$cutvol\left({A}_{k};\widehat{\varvec{S}}\right)=\sum\limits_{j,l\in {A}_{k}}{\widehat{s}}_{jl}.$$

The objective function is defined as the total ANCut.overlap measure, the sum of the between-cluster similarity over the within-cluster similarity ratio,

5 $$ANCut.overlap\left({A}_{1},\dots ,{A}_{K}\right)=\sum\limits_{k=1}^{K}ANCut.overlap\left({A}_{k}\right).$$

For a fixed $K$, the optimal clustering minimizes the total ANCut.overlap measure, as originally proposed by Hidalgo et al. [12]. The difference is that we use $\varvec{S}$ and $\widehat{\varvec{S}}$ computed based on different but partially overlapping sets of individuals. Figure 1 summarizes the flow of the method described above. We refer to this algorithm as *ANCut.overlap*.

### Choosing K

Next, we want to remove the assumption that $K$ is known, selecting $K$ by comparing clustering results for various values of K. Two approaches are adopted and are described below.

1. Average Silhouette method [17].

The average Silhouette method computes the average within- and between-cluster distances between observations for varying numbers of clusters (K). It determines how well each observation lies within its assigned cluster. A high average Silhouette indicates that the observation is well matched to its assigned cluster and poorly matched to neighboring clusters. The Silhouette for an observation is given by

6 $$silh\left(i\right)=\frac{b\left(i\right)-a\left(i\right)}{\text{max}\left\{a\left(i\right), b\left(i\right)\right\}} \text{i}\text{f} \left|{C}_{i}\right|>1\ \text{and} \ silh\left(i\right)=0\ \text{i}\text{f} \left|{C}_{i}\right|=1,$$

where $a\left(i\right)$ is the average Euclidean distance between gene $i$ and all other genes within the same cluster, $b\left(i\right)$ is the lowest average Euclidean distance of gene $i$ to all the genes in any other cluster, of which $i$ is not a member. Note that distance $a\left(i\right), b\left(i\right)$are defined as the inverse of the predicted similarity of two genes, ${\widehat{\varvec{S}}}^{-1}$, instead of the observed distance, ${S}^{-1}$, to incorporate information from regulatory data. The average Silhouette is calculated by taking the mean of the Silhouette values of all observations. We refer to this approach as *ANCut.silh*, application of *ANCut.overlap* using the average Silhouette method to choose the optimal number of clusters K instead of assumming that K is known.

(2) Elbow method [18].

The Elbow method computes the total within-cluster sum of squares (WCSS) for a varying number of clusters (K). WCSS measures the compactness of the clustering and we want WCSS to be small. One should choose the number of clusters so that adding another cluster does not further reduce the total within-cluster sum of squares.

The within-cluster sum of squares for a cluster is given by

7 $$WCSS=\frac{1}{p}\sum\limits_{j=1}^{p}\sum\limits_{l=j+1}{d}_{jl}^{2},$$

where ${d}_{jl}$ is the Euclidean distance between gene $j$ and gene $l$ within the same cluster, calculated based on the inverse of the predicted similarity of two genes, ${\widehat{\varvec{S}}}^{-1}$, instead of the observed distance, ${S}^{-1}$, to incorporate information from regulatory data. For a particular number of clusters $K$, the total $WCSS$ is calculated by summing $WCSS$ over all $K$ clusters. We refer to this approach as *ANCut.elbow*, application of *ANCut.overlap* using the Elbow method to choose the optimal number of clusters K instead of assumming that K is known.

### Computation

To optimize the objective function defined in (2), we adopt the simulated annealing (SA) technique [19]. The algorithm proceeds as follows.

Let $t$ be the iteration index. At iteration $t$, we denote ${A}^{\left(t\right)}=\left\{{A}_{1}^{\left(t\right)},\dots ,{A}_{K}^{\left(t\right)}\right\}$ as the partition (clustering result) and $ANCut.overlap\left(t\right)$ as the value of the objective function.

- Step 1. Randomly initialize ${A}^{\left(0\right)}=\left\{{A}_{1}^{\left(0\right)},\dots ,{A}_{K}^{\left(0\right)}\right\}$.
- Step 2. Based on the partition in iteration $t$, compute ${m}_{k}^{\left(t\right)}$ as the number of gene pairs $\left(j,l\right)$ with $j,l\in {A}_{k}^{\left(t\right)}$ for $k=1,\dots ,K$. Draw two clusters, ${A}_{k\left(-\right)}^{\left(t\right)}$ and ${A}_{k\left(+\right)}^{\left(t\right)}$, from $\left\{{A}_{1}^{\left(t\right)},\dots ,{A}_{K}^{\left(t\right)}\right\}$ with probabilities proportional and inversely proportional to $\frac{{m}_{k}^{\left(t\right)}}{{\sum }_{k=1}^{K}{m}_{k}^{\left(t\right)}}$, respectively. Clusters ${A}_{k\left(-\right)}^{\left(t\right)}$ and ${A}_{k\left(+\right)}^{\left(t\right)}$ are the clusters that we will possibly update in iteration $t+1$.
- Step 3. At iteration $t+1$, draw gene $i$ randomly from cluster ${A}_{k(-)}^{\left(t\right)}$. Set ${A}_{k\left(+\right)}^{\left(t+1\right)}={A}_{k\left(+\right)}^{\left(t\right)}\bigcup \left\{i\right\}$, ${A}_{k(-)}^{\left(t+1\right)}={A}_{k\left(-\right)}^{\left(t\right)}\backslash \left\{i\right\}$, and ${A}_{h}^{\left(t+1\right)} := {A}_{h}^{\left(t\right)}$ for $h\ne k\left(-\right),k\left(+\right)$.
- Step 4. If $ANCut.overlap\left(t+1\right)\le ANCut.overlap\left(t\right),$ accept the update of ${A}^{\left(t+1\right)}$ in step 3. If not, accept the update of ${A}^{\left(t+1\right)}$ in step 3 with probability $\text{exp}\left(-\frac{ANCut.overlap\left(t+1\right)-ANCut.overlap\left(t\right)}{T\left(t+1\right)}\right)$, where $T\left(t\right)=L\text{log}\left(t+1\right)$ is the temperature function with $L$ user-defined as a large number ($L=$ 10,000 in our simulation study and data analysis); and otherwise reject the update in step 3, ${A}^{\left(t+1\right)}={A}^{\left(t\right)}$.
- Step 5. Repeat steps 2–4 until $t$ reaches a pre-specified large number of iterations $B$, e.g. 10,000 in our simulation study and data analysis. Convergence of the SA algorithm has been established in the literature [19]. The value of $B$ is not important, as long as it is large enough [12]. $B=\text{3,000}$ has been shown to be large enough to achieve convergence for datasets of dimension $n=200,p=500,q=500$ [12]. With a similar number of genes measured on more individuals in our simulation study, $B=\text{10,000}$ is selected and should be large enough to achieve convergence. The sufficiency of $B=\text{10,000}$ is further confirmed by additional simulations with $B=\text{20,000}$, the results with $B=\text{20,000}$ and the results with $B=\text{10,000}$ are similar. Because we want the algorithm to determine the optimal number of clusters without pre-specifying $K$, our computation process includes a repeat of the SA algorithm for choosing the optimal number of clusters.

Option 1: Using the average Silhouette method to determine the optimal number of clusters.

- Step 1. Compute clustering algorithm (e.g., SA) for different values of $K$. For instance, $K$ varies from 1 to 10 clusters.
- Step 2. For each $K$, calculate the average Silhouette of all observations (avg.sil).
- Step 3. Plot the curve of avg.sil according to the number of clusters $K$.
- Step 4. The location of the maximum is considered as the appropriate number of clusters.

Option 2: Using the elbow method to determine the optimal number of clusters.

- Step 1. Compute clustering algorithm (e.g., SA) for different values of $K$. For instance, $K$ varies from 1 to 10 clusters.
- Step 2. For each $K$, calculate the total within-cluster sum of squares $WCSS$.
- Step 3. Plot the curve of $WCSS$ according to the number of clusters $K$.
- Step 4. The location of a bend (i.e. max 2nd derivative) in the plot is generally considered as an indicator of the appropriate number of clusters.

### Application to the Framingham Heart Study (FHS)

The Framingham Heart Study (PMID: 474,565, 17,372,189) is a longitudinal study of three generations of participants focused on cardiovascular diseases. It comprises three generations of participants: the Original cohort followed since 1948; the Offspring cohort consisting of their offspring and spouses of the offspring, followed since 1971; and Generation 3 cohort composed of the children and their spouses from the largest Offspring families, enrolled in 2002. The Original cohort enrolled 5,209 men and women who comprised two-thirds of the adult population then residing in Framingham, MA and survivors continue to receive biennial examinations. The Offspring cohort of 5,124 participants (including 3,514 biological offspring), have been examined approximately once every 4–6 years. Generation 3 cohort included 4,095 individuals that have been examined on 4 occasions.

The gene expression profiling has been described in detail by Joehanes et al. [20]. Briefly, fasting peripheral whole blood was collected during clinical visits. Whole blood gene expression was measured by the Affymetrix Human Exon 1.0st Array. The raw data were quantile-normalized and log2 transformed, followed by summarization using Robust Multi-array Average [21]. The data was further adjusted for batch effects and technical covariates, including the first principle component of the expression data, batch effect, and the all-probeset-mean residual. GE profiles for 17,873 genes are available on 5,626 participants from the Offspring cohort who attended exam 8 (2005–2008) and the Generation 3 cohort who attended exam 2 (2008–2011). Out of the 5,626 participants, more than 2,000 participants from the Generation 3 cohort had complete blood count (CBC) values. For the other participants, their CBC values were imputed by PLS (Partial Least Square) and normalized by replacing negative values with zero and adjusting total percentage to 100%. Details have been described elsewhere [20].

The DNA methylation profiling was performed using the Illumina Infinium Human Methylation 450 K BeadChip. Samples were extracted from peripheral whole blood. Methylation profiles, measured in beta values, for 443,298 CpG sites are available on 4,161 participants. There are 21,836 CpG sites with missing values for at least 1 participant, and a total of 1,335 CpG sites contain missing values for more than 1% of the participants. One hundred and thirty-three CpG sites contain missing values for more than 5% of the participants. One CpG site contains missing values for 2,941 participants. This is the maximum proportion of missing we observe from FHS methylation profiles, 2,941/4,161 = 70.7%. To borrow information from methylation regulators and assist in GE clustering, we perform imputation of the missing methylation data. We use methyLImp, a simple and computationally efficient imputation method based on linear regression, to estimate the missing methylation values [22, 23]. The rationale for the approach is that methylation levels exhibit both long- and short-range correlations that can be captured by simple linear regression [24, 25]. The missing values are imputed by iteratively performing linear regression with pseudo-inverse transformation on the available data. We implement the imputation in R using the freely available R-package methyLImp.

It has been reported that the inter-individual variation is heritable and can be mapped as quantitative trait loci (QTLs) [26, 27]. Indeed, mapping studies have revealed that the associations between GE and the QTLs are common and often with large effects. These regulatory variants can act either locally or at a distance to participate in modulating various regulatory epigenetic processes [28]. Therefore, we incorporate genetic variants in addition to methylation as regulators of gene expression, i.e. additional predicting factors in the GE-regulator relationship models. FHS participants were genotyped on the Affymetrix 550 K single nucleotide polymorphism (SNP) array, and imputed to the Haplotype Reference Consortium reference panel release 1.1 using the minimac3 software on the Michigan Imputation Server. Imputed genotypes for 5,608,682 common variants with minor allele frequency (MAF) > = 5% were available from 8481 participants after quality control procedures were applied to omit individuals with low-quality genotypes.

**Fig. 2:**
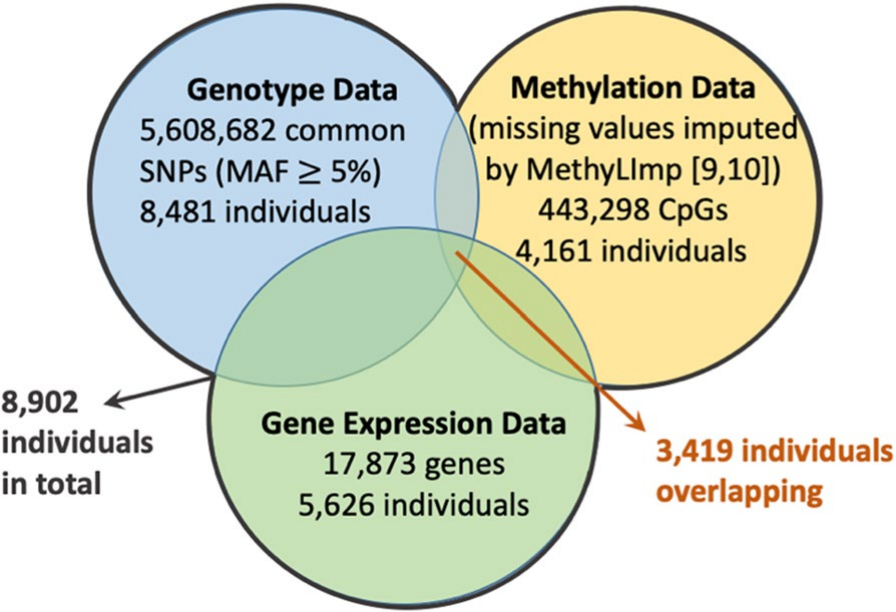
Visualization of FHS data structure

There is an overlap of 3673 individuals between the GE dataset and the methylation dataset, an overlap of 5257 individuals between the GE dataset and the genotype dataset, and an overlap of 3855 individuals between the methylation dataset and the genotype dataset. Overlap between the GE dataset, the methylation dataset, and the genotype dataset includes 3419 individuals. Figure 2 summarizes the structure of the three datasets.

The objective of this analysis is to perform clustering on the genes related to lung function, to gain better insight into genes and pathways affecting pulmonary traits and disease. To select genes with high expression in lung tissues, we extract the expression level, measured as the number of transcripts per million RNA molecules (TPM), in lung tissues for ~ 20k protein-coding genes from the GTEx database (https://gtexportal.org/home/). The trait of interest is the FEV1/FVC ratio. FEV1/FVC ratio represents the proportion of a person’s vital capacity that they are able to expire in the first second of forced expiration (FEV1) to the full, forced vital capacity (FVC). The ratio is often used to diagnose pulmonary diseases such as chronic obstructive pulmonary disease (COPD).

### Identification of GE regulators

To identify GE regulators, we map methylation sites and genetic variants to genes based on their positions. Methylation sites that are located near a gene, i.e. +/- 50 kb around the transcription start site of the gene, and genetic variants that are located near that gene, i.e. +/- 50 kb around the transcription start site or stop site of the gene are identified as candidate regulators of the GE.

### Modeling the GE-methylation-genotype regulation

There is an overlap of 3419 participants between the GE dataset, the methylation dataset, and the genotype dataset. Based on the overlapping individuals, we construct linear mixed effect models of expression for the genes using the methylation sites and the SNPs mapped to the gene, adjusting for the imputed proportions of various cell types such as white blood cell, red blood cell, platelet, lymphocyte, monocyte, eosinophil, basophil as additional covariates in the regression models. In addition, we account for family structure through a kinship matrix when selecting predicting variables through elastic net regularization:

$$\widehat{\varvec{\beta }}=\underset{\varvec{\beta }}{\text{argmin}}\left\{{\left|\left|\varvec{Y}-\varvec{\beta }\varvec{X}\right|\right|}^{2}+\lambda \left(\left(1-\alpha \right){\left|\left|\varvec{\beta }\right|\right|}^{2}+\alpha {\left|\left|\varvec{\beta }\right|\right|}_{1}\right)\right\},$$

where $\lambda >0$ and $0<\alpha <1$ are data-dependent tuning parameters, selected using V-fold cross-validation (V = 5).

Note that we model one gene at a time so that each gene can be predicted by a different set of predictors, i.e. local CpG sites, local SNPs, and cell type proportions. Each gene has its own value for the tuning parameters. In our data analysis, this estimation is implemented using the R package *ggmix* to account for the family correlation structure among the FHS participants using a kinship matrix.

### Assessing GE imputation quality

Because the imputation of GE based on the regulators plays an essential role in the assisted clustering algorithm, we examine the GE imputation quality from the regression model. Imputation quality is measured as the square of the correlation coefficient (${R}^{2}$) between the predicted GE values and the observed GE values. In our simulation study, imputation quality is assessed using overlapping individuals. In the application to FHS data, we fit a linear mixed effect model based on 35% of the 3,419 overlapping individuals and use the model to predict GE for the remaining 65% overlapping individuals. Imputation quality is then assessed based on the predicted GE and the observed GE of these 65% overlapping individuals.

### Assisted clustering with partially overlapping sets of individuals

With the transition matrix estimate $\widehat{\varvec{\beta }}$, we can predict the GE level based on the methylation and SNP observations. Then we calculate the similarity matrix based on the observed GE, $\varvec{S}$, and the predicted GE values, $\widehat{\varvec{S}}$. The last step is to apply the ANCut.overlap algorithm using the computed values of $\varvec{S}$ and $\widehat{\varvec{S}}$.

### Gene set enrichment analysis

With gene clustering results from the methods above, we then proceed with enrichment analysis to better understand the biology behind the clustered gene sets. Gene set analysis methods evaluate pre-specified gene sets for enrichment of modest associations with a disease or trait. Our gene set enrichment analysis (GSEA) starts with a linear regression framework with gene scores as the outcome and the gene cluster membership as the only exposure. This regression tests whether the gene scores in a gene cluster are significantly different from gene scores for genes not included in a specific cluster. Gene scores are defined as the z scores from a transcriptome-wide association study (TWAS) between the expression level of each gene $Y$ and the outcome of interest, after adjusting for potential confounders. We perform TWAS on the genes using FHS samples through linear mixed effect models to account for the familial correlation among related participants, investigating the association between each gene and the outcome FEV1/FVC, adjusting for sex, height, and proportions of various cell types such as white blood cell, red blood cell, platelet, lymphocyte, monocyte, eosinophil, basophil. The gene scores, specifically the z-scores from TWAS, are expected to have a mean of 0 under the null hypothesis that genes are not associated with the trait. Thus, there is no intercept included in the GSEA linear regression. For the clusters enriched with associations with the phenotype of interest, we further analyze the genes in the cluster using the Kolmogorov-Smirnov test to compare the ranks of genes included in these clusters to the ranks of genes not included in these clusters.

### Over-representation analysis (ORA)

For the clusters enriched with associations with the phenotype, the over-representation analysis (ORA) is further performed to examine the degree of over-representation of these genes in various KEGG pathways (https://www.genome.jp/kegg/pathway.html). Over-representation analysis tests if the genes included in these clusters include more genes from the pre-specified KEGG gene sets than expected by chance. For a particular cluster $k, k=1,\dots ,K$, and a particular KEGG pathway, the statistical significance of over-representation is evaluated using a hypergeometric test. A *p*-value < 0.05 is taken as statistical evidence of over-representation.

### Simulation study

We evaluate our novel approach using a simulation study. In our simulations, we consider $n$ individuals, $p$ genes, and $q$ CpG sites. We induce GE clustering structure by imposing clusters on the regulators, i.e. the $q$ CpG sites. $K$ equal-sized methylation clusters are generated. The CpGs/GEs in the same cluster are correlated but the CpGs/GEs in different clusters are uncorrelated. Within each cluster, $\frac{q}{K}$ CpG sites regulate the expression level of $\frac{p}{K}$ genes. Under this data generating structure, there are $K$ clusters of CpGs/GEs. GEs in the $k$th cluster are regulated by methylation of CpGs in the $k$th cluster, $k=1,\dots ,K$. This is equivalent to assuming that the gene expression clusters are due to the component regulated by methylation, rather than the non-regulated component independent of methylation.

#### Scenario I

We use the Framingham Heart Study (FHS) data as a benchmark for data generation in our Scenario I simulation study. The methylation data matrix $\varvec{X}$, of dimension $n\times q$, is first generated from a multivariate normal distribution with mean 0 and correlation matrix

$$\rho=\begin{bmatrix}R_1&\cdots&0\\\vdots&\ddots&\vdots\\0&\cdots&R_K\end{bmatrix}q\times q$$

where the off-diagonal elements of each ${\varvec{R}}_{\varvec{k}}$are fixed to 0.1. Based on FHS data, the median value of the correlation coefficients between methylation of CpGs on the same chromosome is around 0.1, and 90% quantile is around 0.3. We then apply an $\text{expit}\left(\right)$ transform on the $\varvec{X}$ matrix to obtain values in the $\left[\text{0,1}\right]$ range to represent typical methylation beta values.

Gene expression data matrix $\varvec{Y}$ and methylation values are connected with the following regression model:

$$Y=X\beta+\epsilon$$

We define the transition matrix as

$$\beta={\begin{bmatrix}B_1&\cdots&0\\\vdots&\ddots&\vdots\\0&\cdots&B_K\end{bmatrix}}_{q\times p}$$

such that in 95% of the columns (genes) of each ${B}_{k}$, $k=1,\dots ,K,$ 3 out of the $\frac{q}{K}$ elements are non-zero and follow a normal distribution with a mean of 0.5 and a variance of 0.75. In the remaining 5% of the columns (genes), we assign 12 non-zero elements instead of 3 non-zero elements.

We assume that the error matrix follows a multivariate normal distribution:

$$\in\sim N\;\left(\mathbf0\boldsymbol,\boldsymbol\;\underset{}{\boldsymbol\sum}\right),\;\underset{}{\boldsymbol\sum}={\begin{bmatrix}\sigma_1^2&\cdots&\sigma_{1p}\\\vdots&\ddots&\vdots\\\sigma_{p1}&\cdots&\sigma_p^2\end{bmatrix}\text{ }}_{p\times p},\sigma_i^2=\;\frac14\;or\;\frac18,i=1,\dots,p.$$.

For each $i=1,\dots ,p$, most of the ${\sigma }_{ij}$’s are $0$ with $5{\%}$ of ${\sigma }_{ij}>0$ with correlation coefficient following a normal distribution with mean 0.05 and variance of 0.02, and $2{\%}$ of ${\sigma }_{ij}<0$ with correlation coefficient following a normal distribution with mean − 0.05 and variance of 0.02. This results in the following: only 5% of the genes can be well predicted by methylation because (1) the number of methylation predictors is 12 instead of 3; (2) the variance of the error term is 1/8 instead of 1/4. Imputation quality for the majority of the genes will be poor. This reflects what we observe in Framingham Heart Study (FHS) data.

We first assume that there is no missing data, i.e. we have access to GE and methylation measures for all individuals. This would allow the application of the method developed by Hidalgo et al. [12]. We then generate data with varying degrees of missingness, i.e. we have access to a subset of individuals with GE data and another subset with methylation data and we then model the regulation based on the overlapping individuals. In the simulation analysis, let $pcnt$ determine the proportion of overlapping individuals. We consider $pcnt*n$ overlapping individuals. The proportions of missing in methylation and GE are $f*\left(1-pcnt\right)$ and $\left(1-f\right)*\left(1-pcnt\right)$ respectively, where $f$ is the proportion of the non-overlapping individuals with available GE data. We have explored values of $f=0.3, 0.5,$ and 0.7 in the simulation analysis.

#### Scenario II

To evaluate the robustness of the algorithm, we consider a scenario where we incorrectly identify the predictors.

To simulate this scenario, the construction of the methylation matrix and the regression model remains the same as in Scenario I. There are certain modifications to simulate the transition matrix and the error matrix.

We define the transition matrix

$$\beta={\begin{bmatrix}B_1&\cdots&0\\\vdots&\ddots&\vdots\\0&\cdots&B_K\end{bmatrix}}_{q\times p}$$

such that there are only 2 non-zero elements following N(0.5, 0.75) distribution in each of the block ${B}_{k}$, $k=1,\dots ,K$.

We define the error matrix to follow a multivariate normal distribution:

$$\in\sim N\;\left(\mathbf0\boldsymbol,\boldsymbol\;\underset{}{\boldsymbol\sum}\right),{\;\underset{}{\boldsymbol\sum}}={\begin{bmatrix}\sigma_1^2&\cdots&\sigma_{1p}\\\vdots&\ddots&\vdots\\\sigma_{p1}&\cdots&\sigma_p^2\end{bmatrix}\text{ }}_{p\times p},\sigma_i^2=\frac12,i=1,\dots,p.$$

However, for each $i$, 50% of ${\sigma }_{ij}\mathrm{'s}$ are positive with correlation coefficient following$N\left(0.30, 0.10\right)$ and 40% are negative with correlation coefficient following $N\left(-0.30, 0.10\right)$.

Under this scenario, the correlation structure of the gene expression is dominated by the correlation embedded in the error matrix rather than from the measured regulators.

To evaluate the accuracy of the clustering algorithm, we consider the adjacency matrix

$$\varvec{C}={\left({c}_{jl}\right)}_{p\times p}, {c}_{jl}=\left\{\begin{array}{c}1, if\ gene\ g\ and\ gene\ l\ are\ within\ the\ same\ cluster\\ -1, if\ gene\ g\ and\ gene\ l\ are\ within\ different\ clusters\end{array}\right.$$

The accuracy is measured as the degree of consistency between the true adjacency matrix ${\varvec{C}}_{\varvec{T}}$ and the estimated adjacency matrix $\widehat{\varvec{C}}$, defined by

8 $${M}_{accuracy}=\frac{{\sum }_{1\le i\le p,1\le j\le p}{\left({\varvec{C}}_{\varvec{T}}\odot \widehat{\varvec{C}}\right)}_{ij}}{{\sum }_{1\le i\le p,1\le j\le p}{\left({\varvec{C}}_{\varvec{T}}\odot {\varvec{C}}_{\varvec{T}}\right)}_{ij}},$$

where $\odot$ is the component-wise product. A larger value suggests a higher accuracy.

## Results

### Application to FHS

We apply the ANCut.overlap clustering with the average Silhouette method and the elbow method to the Framingham Heart Study (FHS) data. Table 1 summarizes the demographic and clinical characteristics of the FHS participants in the GE dataset, the DNA methylation dataset, and the genotype dataset.

**Table 1:** Demographic and clinical characteristics of the FHS participants

Characteristics	Datasets		
	GE dataset	Methylation dataset	Genotype dataset
Age (years), mean ± sd	44 ± 9.1	43 ± 8.7	45 ± 9.4
Sex, N (%)			
Male	2583 (45.9%)	1925 (46.3%)	3490 (47.0%)
Female	3043 (54.1%)	2236 (53.7%)	3940 (53.0%)
Height (inches), mean ± sd	66.4 ± 3.8	66.2 ± 3.8	66.3 ± 3.8
Weight (pounds), mean ± sd	177.3 ± 41.5	175.9 ± 40.6	176.5 ± 41.3
BMI, mean ± sd	28.2 ± 5.7	28.1 ± 5.5	28.1 ± 5.6
Smoking, N (%)			
Non-smoker	2716 (48.3%)	1907 (45.8%)	2851 (45.8%)
Former smoker	2400 (42.7%)	1901 (45.7%)	2811 (45.1%)
Current smoker	509 (9.0%)	353 (8.5%)	567 (9.1%)
Cigarettes per day, mean ± sd	1.3 ± 4.9	1.2 ± 4.6	1.3 ± 4.9
FEV1 (observed / predicted), mean ± sd	0.98 (0.15)	0.98 (0.16)	0.98 (0.16)
FVC (observed / predicted), mean ± sd	1.02 (0.14)	1.02 (0.14)	1.02 (0.14)
FEV1/FVC (observed / predicted), mean ± sd	0.95 (0.09)	0.95 (0.09)	0.95 (0.09)

A total of 4152 genes with lung tissue TPM > = 1 in 50% of the GTEx samples are selected. GE profiles for these 4152 genes are available on 5626 FHS participants. A figure that shows the distribution of these 4152 genes in the 5626 FHS individuals is available in supplementary materials.

For each of the 4152 genes, we identify the methylation sites that are located near that gene, i.e. +/- 50 kb around the transcription start site of the gene. In total 207,865 CpG sites are mapped to the 4152 genes. Methylation profiles, measured in beta values, of these 20,7865 CpG sites are available on 4161 FHS participants. A figure that shows the distribution of the distance, in base pair, between the methylation sites to their mapped genes is available in the supplementary materials. In addition, the Fig. 5 that shows the distribution of beta values of the methylation sites in the 4161 FHS participants can also be found in supplementary materials.

To predict gene expression most accurately, we include genetic variants as additional predictors. In total 1,103,723 common SNPs with minor allele frequency (MAF) > = 5% are mapped to the 4,152 genes. Genotype data for these 1,103,723 SNPs are available on 8,481 participants.

Based on the 3419 overlapping individuals, we model the regulation relationship between GE and methylation together with genotype. Because we perform penalized regression, the majority of variables are not selected to have any effect on GE. For many of the genes, the optimal fit of the penalized regression is achieved when only the cell type proportions have non-zero coefficients – none of the local CpG sites or the local SNPs is estimated to have any regulating effect on the expression of those genes. Out of the 4152 genes modeled, 2179 genes are predicted by at least 1 CpG site or 1 SNP in the penalized regression. Because we want to incorporate the regulation information from CpGs and SNPs to assist in GE clustering, the following co-expression analysis is performed only on these 2179 genes. In this way, we investigate the GE clustering structure of genes that are regulated by the measured methylation and genotype.

With ANCut.overlap clustering, the Silhouette method determines that the best fit is achieved with 13 clusters, whereas the elbow method achieves optimal fit with 10 clusters. Considering that there are a large number of genes, we allow for more clusters and select $K=13$ to be used in the following analyses. Table 2 shows the degree of consistency between the adjacency matrices according to different clustering algorithms. As a comparison, we also perform ANCut.subset clustering with 13 clusters and K-means clustering with 13 clusters.

**Table 2:** Consistency between different clustering algorithms on FHS

Consistency of Adjacency Matrix b/w Clustering Results	ANCut.silh (K = 13)	ANCut.elbow (K = 10)	ANCut.subset (K = 13)	K-means (K = 13)
ANCut.silh	1	82.0%	73.1%	80.6%
ANCut.elbow	82.0%	1	69.3%	83.2%
ANCut.subset	73.1%	69.3%	1	68.9%
Kmeans	80.6%	83.2%	68.9%	1

A closer examination of the ANCut.overlap clustering with the Silhouette method, ANCut subset clustering with $K=13$, and the K-means with $K=13$ shows that the ANCut.overlap clustering and ANCut.subset clustering tend to give more balanced clusters in terms of the number of genes included in each cluster. Table 3 shows the number of genes assigned to each cluster, comparing the three clustering approaches. Assignment by K-means provides clusters with more varied numbers of genes per cluster.

**Table 3:** Comparison of gene cluster size (number of genes) across different clustering approaches

Number of genes in cluster	1	2	3	4	5	6	7	8	9	10	11	12	13
ANCut.silh	255	127	147	133	134	221	183	124	176	117	286	169	107
ANCut.subest with K = 13	205	163	248	128	176	84	192	94	181	156	188	141	223
K-means with K = 13	323	173	102	358	148	60	39	283	101	50	254	17	271

Please note that the cluster number assignment is arbitrary. There is no 1-to-1 correspondence between the ANCut.silh clustering assignment, ANCut.subset clustering assignment, and the K-means clustering assignment

Next, we want to check if the ANCut.overlap clustering minimizes the objective function, i.e. the sum of the between-cluster similarity over the within-cluster similarity ratio. Table 4 presents the $ANCut.overlap$ measure of each of the 13 clusters ${A}_{1},\dots ,{A}_{13}$, defined in (2)–(4),and the objective function, the sum of all the clusters $ANCut.overlap={\sum }_{k=1}^{13}ANCut.overlap\left(k\right)$, comparing the three clustering algorithms ANCut.silh, ANCut.subset, and K-means. The $ANCut.overlap\left({A}_{k}\right)$ measures are obtained from the ANCut.overlap clustering algorithm, we sum up the $ANCut.overlap\left({A}_{k}\right)$ measures based on different clustering partitions assigned by the three clustering approaches.

**Table 4:** Comparison of $ANCut.overlap\,\left({A}_{k}\right)$ of each cluster across different clustering approaches

	1	2	3	4	5	6	7	8	9	10	11	12	13	sum
ANCut.silh	2.6	3.4	3.4	3.4	3.7	3.6	2.9	3.6	2.9	3.9	2.2	3.4	4.1	43.2
ANCut.subest with K = 13	7.4	8.0	9.1	7.5	7.9	7.0	7.0	6.7	8.0	6.7	7.1	8.1	8.3	98.8
K-means with K = 13	1.7	2.9	4.0	1.4	3.3	6.6	8.3	2.0	4.3	6.5	2.0	21.3	1.9	66.2

ANCut.silh clustering gives lower $ANCut.overlap$ value than K-means does, 43.2 < 66.2. This confirms that the ANCut.overlap clustering is indeed minimizing the sum of the between-cluster similarity over the within-cluster similarity ratio. Surprisingly, ANCut.subset algorithm gives the highest $ANCut.overlap$ value. When the number of individuals with complete overlap of omics dataset is limited, ANCut.subset performs worse than K-means does, in terms of minimizing the sum of the between-cluser similarity overth within-cluster similarity ratio.

**Fig. 3:**
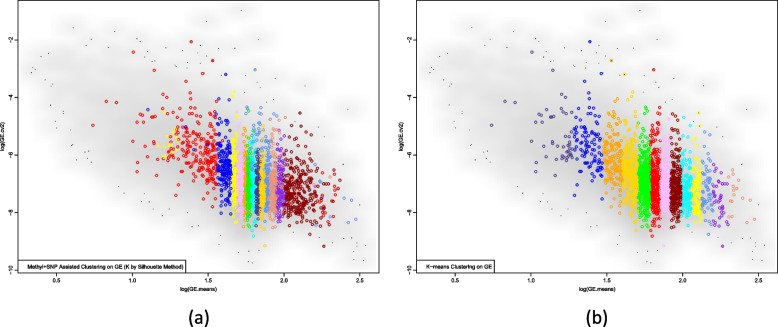
Comparison of cluster assignment across different clustering approaches. Panel (**a**): cluster assignment by ANCut.silh clustering incorporating methylation and SNPs data; panel (**b**): cluster assignment using K-means clustering. The x-axis shows the mean of gene expression level averaged over FHS samples, log transformed; the y-axis shows the coefficient of variation of gene expression level, log-transformed

Figure 3 compares the cluster assignment of each gene.

Comparing the results of the K-means clustering and the ANCut.silh clustering, K-means classifies the genes solely based on the means of the gene expression levels; however, ANCut.overlap clustering classifies the genes based on not just the means. For example, the cluster colored in dark blue spreads from 1.0 to 1.5 on the x-axis, which overlaps with the cluster colored in red and yellow. Also, the cluster colored in red spreads from the minimum value on the x-axis to almost the value maximum on the x-axis.

**Fig. 4:**
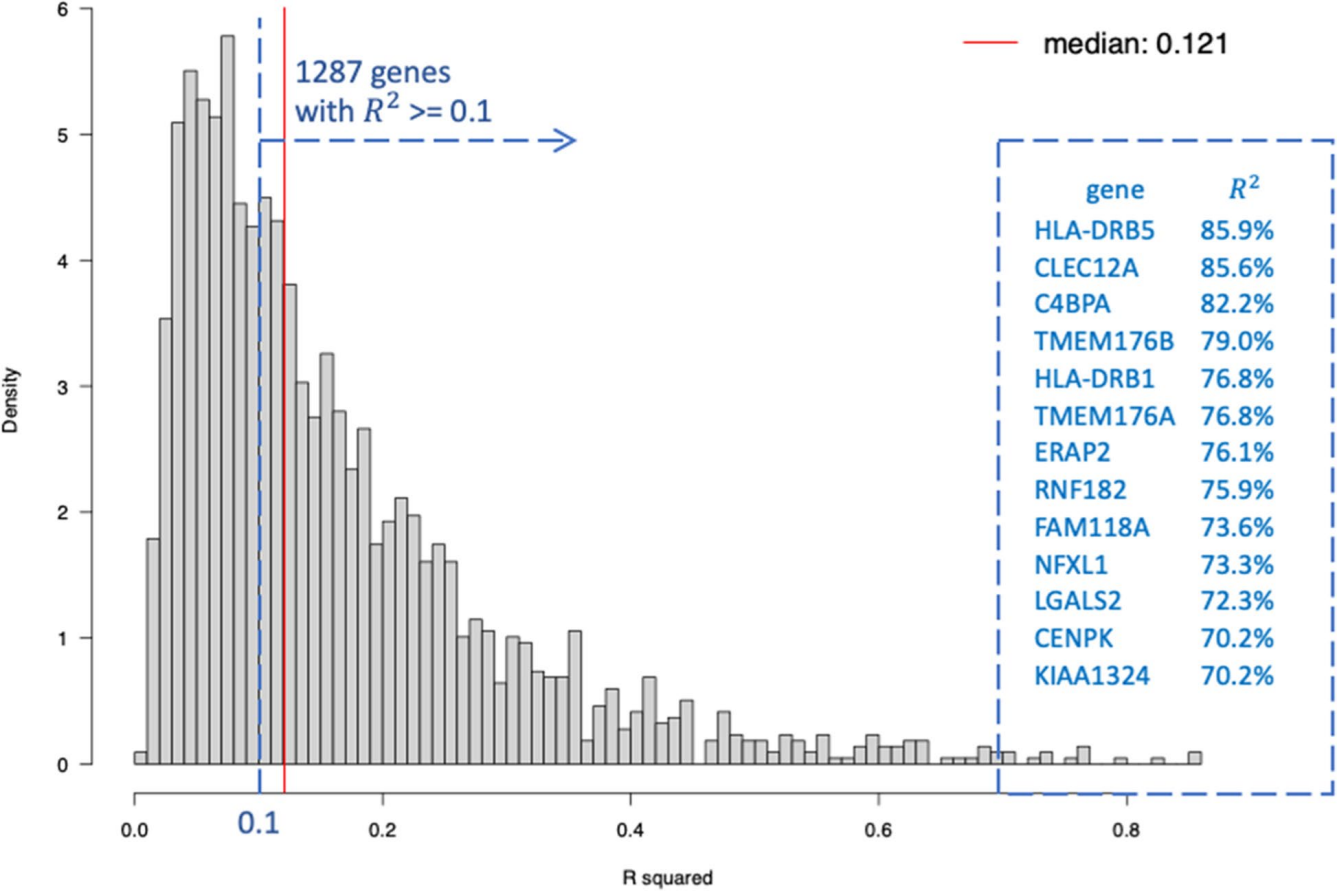
Distribution of the GE imputation quality evaluated in 65%*3,419=2,222 FHS individuals

Note that imputation is an important step in the ANCut.overlap clustering. We assess the imputation quality of GE using local methylation, local SNPs, and cell type proportions in FHS. Figure 4 shows the distribution of the imputation quality measured as ${R}^{2}$ of the genes.

Further looking at the 13 genes highlighted in Fig. 4 with ${R}^{2}$ higher than 0.7, we find that our proposed approach ANCut.overlap assigns these genes into 4 different clusters, whereas K-means assigns these genes into 8 different clusters. This implies that our approach, while borrowing information from methylation and genotype, is more likely to detect the correlation structure across GEs.

Next, we proceed to the gene set enrichment analysis (GSEA), starting with a linear regression model with association gene scores as the outcome and gene cluster membership as the only exposure, no intercept included. Table 5 shows the GSEA linear model result.

**Table 5:** GSEA linear model based on ANCut.silh clustering result

	ANCut.silh clustering			K-means clustering		
	Estimate	Std error	*P* value	Estimate	Std error	*P* value
Cluster 1	0.102	0.076	0.180	0.039	0.068	0.561
Cluster 2	0.032	0.108	0.766	0.078	0.092	0.397
Cluster 3	0.014	0.100	0.889	-0.287	0.120	0.017
Cluster 4	0.171	0.106	0.105	0.111	0.064	0.085
Cluster 5	0.076	0.105	0.469	-0.281	0.100	0.005
Cluster 6	0.010	0.082	0.906	0.075	0.157	0.631
Cluster 7	-0.195	0.090	0.030	-0.103	0.195	0.597
Cluster 8	0.084	0.109	0.441	-0.095	0.072	0.188
Cluster 9	0.045	0.092	0.620	0.193	0.121	0.112
Cluster 10	-0.108	0.113	0.335	0.038	0.172	0.824
Cluster 11	-0.235	0.072	0.001	-0.104	0.076	0.172
Cluster 12	-0.064	0.094	0.493	-0.380	0.295	0.198
Cluster 13	0.007	0.118	0.949	0.010	0.074	0.888

**Fig. 5:**
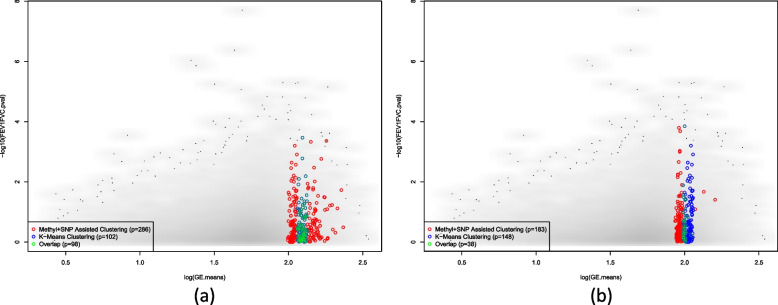
Genes of the lowest and the 2^nd^ lowest GSEA *p*-value cluster membership. Panel (**a**): overlap (green circles) between genes assigned to cluster 11 from ANCut.silh clustering (red circles) and genes assigned to cluster 3 from K-means clustering (blue circles); Panel (**b**): overlap (green circles) between genes assigned to cluster 7 from ANCut.silh clustering (red circles) and genes assigned to cluster 5 from K-means clustering (blue circles). x-axis shows the mean of gene expression level averaged over FHS individuals, log-transformed; y-axis shows the -log of the *p*-value of association between gene expression and FEV1/FVC

We then take a closer look at genes assigned to the clusters with the lowest *p*-values, e.g. clusters 11 and 7 from ANCut.silh clustering assignment, clusters 5 and 3 from K-means clustering assignment. Figure 5 shows that there is a substantial proportion of overlap between the genes in these clusters.

Kolmogorov-Smirnov (KS) test is performed on clusters 11 and 7 from ANCut.silh clustering. The KS *p* values of clusters 11 and 7 are 0.013 and 0.090 respectively. Figures that show KS test details are available in supplementary materials.

Next, we perform over-representation analysis (ORA) to examine the degree of over-representation of the genes in several KEGG pathways. ORA *p*-values are calculated for clusters 11 and 7, the two clusters with the lowest GSEA enrichment *p*-values. As a comparison, ORA is also performed on clusters 6 and 9, the two clusters with the least significant GSEA enrichment *p*-value and with a comparable number of genes. The significance of the over-representation analysis is summarized in Table 6.

**Table 6:** ORA *p* values of genes compared with KEGG pathways

	Immune (*n* = 337)	Diabetes (*n* = 93)	Lung (*n* = 126)	COVID-19 (*n* = 26)	Liver (*n* = 221)	Colorectal (*n* = 49)	Skin (*n* = 47)	Neuro-degeneration (*n* = 139)	Kidney (*n* = 93)
No. 11	0.018	0.054	5.3e-04	9.9e-04	5.8e-03	0.468	0.884	0.034	0.015
No. 7	0.065	0.085	0.058	0.375	2.0e-04	0.111	0.566	4.7e-03	0.152
No. 6	0.546	0.340	0.640	0.758	0.666	0.565	0.426	0.556	0.613
No. 9	0.996	0.952	0.951	0.890	0.923	0.917	0.905	0.889	0.952

Cluster 11 is over-represented in the immune-related, diabetes, lung, COVID-19, liver, neuro-degeneration, and kidney disease pathways. Cluster 7 is over-represented in the immune-related, diabetes, lung, liver, and neuro-degeneration pathways. Clusters 6 and 9 are not over-represented in any of the pathways of interest.

### Simulation studies

#### Scenario I

Table 7 summarizes the GE imputation quality under different simulation settings described for scenario I based on 100 simulation replicates. As shown in Table 7, the maximum imputation quality can be as high as 0.7 under certain settings but the median imputation quality remains as low as 0.1. This is due to how we generate the data – only 5% of the genes can be well predicted, whereas the imputation quality is poor for the remaining 95% of genes, resembling what was observed in the FHS data.

Simulation study results with higher off-diagonal elements of ${R}_{k}$, $\text{c}\text{o}\text{r}\text{r}=0.3$, in the correlation matrix $\rho$ of the methylation data, as well as simulation study results with other values of $f$, 0.3 and 0.7, the proportion of the non-overlapping individuals with available GE data are summarized in the supplementary materials.

**Table 7:** Imputation quality under various simulation coefficient settings with fixed $p=500$ and $pcnt=1/3$

Median ${R}^{2}$ (range)		$q$					
		200	400	500	600	800	1000
$n$	300	0.056 (1e-06, 0.65)	0.039 (3e-07, 0.53)	0.036 (1e-07, 0.42)	0.036 (1e-06, 0.52)	0.033 (5e-07, 0.56)	0.039 (4e-07, 0.56)
	500	0.065 (3e-06, 0.66)	0.059 (2e-06, 0.53)	0.053 (2e-07, 0.6)	0.043 (9e-07, 0.52)	0.044 (3e-06, 0.57)	0.041 (2e-06, 0.45)
	1000	**0.099** **(1e-08, 0.72)**	0.073 (4e-09, 0.6)	0.071 (1e-05, 0.57)	0.066 (5e-07, 0.6)	0.069 (3e-06, 0.53)	0.063 (6e-05, 0.5)
	1200	**0.111** **(6e-07, 0.75)**	0.083 (1e-07, 0.6)	0.084 (1e-04, 0.58)	0.087 (2e-06, 0.63)	0.076 (7e-10, 0.57)	0.06 (2e-06, 0.57)
	1500	**0.131** **(8e-05, 0.78)**	0.1 (2e-05, 0.63)	0.097 (2e-06, 0.64)	0.088 (6e-05, 0.55)	0.074 (3e-06, 0.57)	0.069 (1e-08, 0.52)

Imputation quality under varying proportions of overlapping individuals ($pcnt$) is summarized in the supplementary materials

Summary statistics of clustering accuracy are presented in Table 8. Median values are computed based on 100 replicates.

**Table 8:** Clustering accuracy under various simulation coefficient settings with fixed $p=500$

Parameters				Median Accuracy Measure ${M}_{accuracy}$					
$n$	$q$	$K$	$pcnt$	ANCut	ANCut.subset	ANCut.overlap	ANCut.silh	ANCut.elbow	K-means
300	200	3	1/3	52.0%	41.7%	43.5%	19% (K = 2)	18.7% (K = 2)	23.9%
1500	200	3	1/3	69.6%	61.8%	63.0%	63% (K = 3)	62.6% (K = 3)	36.3%
300	500	3	1/3	55.0%	38.4%	41.1%	39.9% (K = 3)	41.1% (K = 3)	30.3%
1500	500	3	1/3	64.6%	57.1%	58.3%	58.3% (K = 3)	58.6% (K = 3)	25.3%
300	1000	3	1/3	62.6%	42.8%	44.7%	43.7% (K = 3)	45.3% (K = 3)	44.1%
**1500**	**1000**	**3**	**1/3**	**71.5%**	**64.7%**	**67.2%**	**67.1% (K = 3)**	**67.6% (K = 3)**	**28.6%**
300	200	5	1/3	66.0%	48.5%	51.8%	10.9% (K = 2)	54.6% (K = 5)	45.0%
1500	200	5	1/3	73.7%	70.3%	72.3%	72.2% (K = 5)	70.5% (K = 6)	31.3%
300	500	5	1/3	61.5%	51.7%	52.6%	46% (K = 4)	33.6% (K = 3)	46.9%
1500	500	5	1/3	67.3%	63.3%	67.9%	66.9% (K = 5)	67.7% (K = 5)	22.6%
300	1000	5	1/3	70.2%	51.2%	55.0%	10.9% (K = 2)	10.5% (K = 2)	53.6%
**1500**	**1000**	**5**	**1/3**	**76.1%**	**68.8%**	**74.9%**	**73.7% (K = 5)**	**71.4% (K = 5)**	**38.4%**
300	500	3	1/5	59.0%	28.4%	30.9%	17.4% (K = 2)	16.8% (K = 2)	34.6%
1500	500	3	1/5	66.7%	56.3%	58.3%	58.3% (K = 3)	58.3% (K = 3)	24.4%
300	1000	5	1/5	67.1%	41.2%	47.6%	6.2% (K = 2)	6.7% (K = 2)	35.6%
**1500**	**1000**	**5**	**1/5**	**75.2%**	**60.1%**	**66.6%**	**66.8% (K = 5)**	**66.9% (K = 5)**	**23.7%**
300	500	3	1/9	60.9%	20.4%	26.3%	22.7% (K = 3)	25% (K = 3)	45.1%
1500	500	3	1/9	65.9%	49.6%	53.2%	53.2% (K = 3)	53.3% (K = 3)	34.1%
300	1000	5	1/9	66.9%	37.7%	40.9%	4% (K = 2)	5.2% (K = 2)	40.8%
**1500**	**1000**	**5**	**1/9**	**76.9%**	**55.9%**	**56.4%**	**56.2% (K = 5)**	**55.9% (K = 5)**	**25.5%**

Column definitions:

*ANCut* uses Hidalgo’s assisted clustering approach to cluster gene expression, with no missing data [12]. This serves as the “gold standard” as we compare various clustering approaches because this approach uses the largest amount of data – the entire gene expression data matrix and methylation data matrix in Fig. 1. The true number of clusters $K$ is assumed to be known

*ANCut.subset* also uses Hidalgo’s assisted clustering approach to cluster gene expression but uses only the overlapping individuals that have both GE and methylation data. The true number of clusters $K$ is assumed to be known

ANCut.overlap uses the proposed approach assuming only a subset of the data is available, i.e. the $X$ and $Y$ matrices in Fig. 1. With the $\left(pcnt*n\right)$ overlapping individuals, we can construct a regression model between GE (${Y}^{O}$) and the methylation regulators (${X}^{O}$) to improve GE clustering. The true number of clusters $K$ is assumed to be known

*ANCut.silh* uses the proposed approach (ANCut.overlap) with the Silhouette method to select the optimal number of clusters

*ANCut.elbow* uses the proposed approach (ANCut.overlap) with the Elbow method to select the optimal number of clusters

*K-means* uses K-means method to cluster GE, using only the $Y$ matrix (with missing data) in Fig. 1. The true number of clusters $K$ is assumed to be known

Clustering accuracy under additional simulation coefficient settings is summarized in the supplementary materials

#### Scenario II

Table 9 summarizes the imputation quality with various parameter settings under the poor-imputation quality scenario based on 100 replicates. The number of genes $p=500$, the correlation coefficient between methylation of CpGs $\text{c}\text{o}\text{r}\text{r}=0.1$, and varying the proportion of the non-overlapping individuals with available GE data $f=0.5$ remain fixed.

**Table 9:** Imputation quality under the poor imputation quality scenario with various simulation coefficient settings and fixed $pcnt=1/3$

Median ${R}^{2}$ (range)		$q$					
		200	400	500	600	800	1000
$n$	300	0.011 (3e-07, 0.21)	0.013 (6e-08, 0.20)	0.009 (6e-09, 0.21)	0.008 (3e-07, 0.18)	0.008 (2e-07, 0.18)	0.009 (4e-09, 0.21)
	500	0.013 (6e-11, 0.25)	0.011 (3e-07, 0.19)	0.007 (1e-09, 0.22)	0.009 (4e-08, 0.20)	0.010 (5e-07, 0.29)	0.007 (9e-08, 0.16)
	1000	0.016 (2e-09, 0.33)	0.009 (1e-08, 0.25)	0.013 (5e-07, 0.25)	0.009 (1e-07, 0.27)	0.011 (2e-07, 0.21)	0.007 (3e-09, 0.15)
	1200	0.022 (5e-07, 0.28)	0.014 (7e-08, 0.22)	0.014 (2e-07, 0.21)	0.011 (3e-10, 0.19)	0.011 (4e-08, 0.20)	0.012 (3e-08, 0.23)
	1500	0.023 (4e-07, 0.31)	0.019 (2e-07, 0.31)	0.014 (2e-07, 0.25)	0.011 (3e-08, 0.29)	0.012 (2e-07, 0.25)	0.008 (4e-07, 0.23)

Imputation quality under additional proportions of overlapping individuals ($pcnt$) is summarized in the supplementary materials

Comparison of clustering accuracy between different clustering approaches is presented in Table 10. Median values are computed based on 100 replicates. The number of genes $p=500$ remains fixed.

**Table 10:** Clustering accuracy under the poor imputation quality scenario with various simulation coefficient settings and fixed $pcnt=1/3$

Parameters			Median Accuracy Measure ${M}_{accuracy}$					
$n$	$q$	$K$	ANCut	ANCut.subset	ANCut.overlap	ANCut.silh	ANCut.elbow	K-means
500	200	3	43.1%	23.9%	22.4%	8.1% (K = 2)	8.1% (K = 2)	11.5%
1500	200	3	47.6%	40.1%	40.2%	40.2% (K = 3)	40.9% (K = 3)	11.4%
3000	200	3	62.9%	54.8%	54.1%	54.4% (K = 3)	54.1% (K = 3)	12.1%
500	500	3	41.0%	24.1%	23.1%	8.6% (K = 2)	8.7% (K = 2)	11.6%
1500	500	3	49.8%	37.4%	38.1%	37.9% (K = 3)	37.9% (K = 3)	11.4%
3000	500	3	52.4%	50.4%	50.9%	50.5% (K = 3)	51.4% (K = 4)	11.4%
500	1000	3	42.7%	16.2%	20.2%	4.5% (K = 2)	5.6% (K = 2)	11.3%
1500	1000	3	52.3%	44.6%	46.2%	46.2% (K = 3)	45.9% (K = 3)	11.5%
3000	1000	3	57.0%	47.2%	46.5%	52% (K = 4)	47% (K = 3)	11.6%
500	200	5	48.8%	40.2%	40.2%	4.1% (K = 2)	2.7% (K = 2)	36.3%
1500	200	5	63.3%	54.2%	53.1%	53.5% (K = 5)	6.1% (K = 2)	36.3%
3000	200	5	63.1%	63.7%	64.7%	63.4% (K = 5)	62.7% (K = 6)	36.5%
500	500	5	51.7%	40.2%	40.8%	3.8% (K = 2)	4.5% (K = 2)	36.4%
1500	500	5	58.9%	52.3%	54.7%	44.4% (K = 4)	48.8% (K = 5)	36.2%
3000	500	5	65.1%	57.2%	58.7%	58.1% (K = 5)	58.4% (K = 5)	36.4%
500	1000	5	49.6%	39.1%	38.9%	2.2% (K = 2)	1.8% (K = 2)	36.3%
1500	1000	5	62.6%	51.3%	53.9%	44.7% (K = 4)	52.8% (K = 5)	36.4%
3000	1000	5	68.1%	61.4%	60.6%	60.2% (K = 5)	59.5% (K = 5)	36.3%

Please refer to Table 8 for an explanation of the column definitions

Clustering accuracy under the poor imputation quality scenario with $pcnt=1/5$ or $1/9$ are summarized in the supplementary materials

## Discussion

We proposed a novel approach for clustering GE with the assistance of regulatory data that allowed for different but partially overlapping sets of individuals to be included in different omics data. We evaluated our approach by simulations and an application to FHS data. We found that the proposed approach showed competitive performance in terms of accuracy compared to the alternative K-means method, especially when the clustering structure was due mostly to the regulated component, rather than the non-regulated component. Compared to the ANCut approach applied on the full simulated dataset without missing data, the proposed approach assuming only a subset of observations were part of both omics datasets showed less accurate but satisfactory performance, because ANCut could only be applied when there was no missingness [12]. In real data analysis, we would not have access to the full data. Instead, we can apply the ANCut approach to only the overlapping individuals that have both omics data, a subset of the full simulated dataset. Compared to this approach, our proposed approach showed more accurate performance when the clustering structure was due mostly to the regulated component.

The performance of the proposed approach depended on the strength of the GE-regulator relationship, the degree of missingness, data dimensionality, sample size, the number of clusters, and other factors. Across the many various simulation settings presented in Table 8, the proposed method was observed to have competitive performance in terms of accuracy. Specifically, clustering results of the proposed method (column *ANCut.overlap*) were almost always more accurate compared to that of K-means clustering (column *K-means*), and were always more accurate than that of ANCut method applied to only the individuals that have both omics data (column *ANCut.subset*). The accuracy of the novel approach was not far from the results obtained by ANCut method assuming all samples were available in both omics datasets (column *ANCut*). Note that the performance of both the proposed approach and ANCut decayed as the number of CpG sites unassociated with GE increased. This was expected because both approaches involved estimating the regulation relationship. In our simulation study, when we generated data, we assumed a fixed number of CpG regulators for each gene, i.e. the number of non-zero elements in the columns of the transition matrix $\beta$. Increasing $q$, the number of methylation sites, did not help but only added more noise in the regression model when we estimated the transition matrix. This was consistent with what we presented in Table 7, the imputation quality decreased as $q$ increased, given the other parameters. The decrease in the imputation quality resulted in a decrease in the clustering accuracy.

For the same reason, we observed that the clustering accuracy increased as the sample size $n$ or the overlap proportion $pcnt$ increased. Particularly, we wanted to emphasize the impact of the overlap proportion $pcnt$. As presented in Table 8, when $pcnt$ was as high as 1/3, the accuracy of the proposed approach was often quite close to that of ANCut without missingness, higher than that of ANCut applied on only the subset individuals with both omics data, and even higher than that of K-means. Remarkably, with $n=1500,q=1000,K=5,pcnt=1/3$, the median accuracy of the proposed approach was 74.9%, only 1.2% lower than that of ANCut, 76.1%, and 6.1% higher than ANCut.subset, 68.8%, whereas K-means gave a median accuracy of 38.4%. As $pcnt$ decreased, the difference between the accuracy of the proposed approach and ANCut increased, and the difference between the accuracy of the proposed approach and K-means decreased. When $pcnt$ was as low as 1/9, occasionally K-means outperformed the proposed approach. For example, with $n=300,q=500,K=3,pcnt=1/9,$ the median accuracy of the proposed approach was 26.3%, lower than that of K-means 45.1%.

We did not see a clear trend in the accuracy of the proposed approach as we varied $K.$ However, increasing $K$ made it more difficult to select the correct number of clusters for both the Silhouette method and the Elbow method [17, 18]. As presented in Table 8, when $K=3$, most often, both the Silhouette method and the Elbow method were able to correctly identify the number of clusters. However, when $K=5$, for many of the simulation replicates, the $K$ that achieved the optimal fit in the Silhouette method or the Elbow method was not the true number of clusters used in generating the simulated data. However, the probability of identifying the correct $K$ increased as the sample size $n$ increased. For example, with $K=5$ in the bottom two rows of Table 8, when $pcnt$ was as low as 1/9 and $q$ was as high as 1000, with $n=300$, both methods failed to identify the correct $K$ thus providing inaccurate clustering results (median ${M}_{accuracy}$ of 4% and 5.2%); but with $n=1500,$ both approaches were able to select the correct $K$ thus providing comparably accurate clustering results (median ${M}_{accuracy}$ of 56.2% and 55.9%). It is expected that the accuracy was low when the algorithm selected the wrong number of clusters ($K$) – the true adjacency matrix and the estimated adjacency matrix could hardly be similar if they had a different number of clusters embedded. When the algorithm selected the correct $K,$ we observed very similar accuracy results between the columns ANCut.silh and ANCut.elbow. This demonstrated the stability of our proposed approach because once ANCut.silh and ANCut.elbow selected the optimal $K$, the remaining part of the algorithms are identical to that of ANCut.overlap.

Although our proposed approach depended on the GE-regulator regulation relationship, we wanted to emphasize the robustness of the approach. Specifically, as presented in Table 10, under the poor imputation quality scenario where the correlation structure of GE was dominated by the correlation embedded in the error matrix rather than from the measured regulators, the accuracy of the proposed approach was almost always higher than that of K-means, and similar to that of ANCut.subset. But the identification of the correct $K$ was further challenged with the decrease in the strength of the regulation relationship – even when $n$ was large and $q$ was small, both the Silhouette and Elbow methods performed poorly in terms of finding the correct $K$. Yet, again, once the algorithm selected the correct $K$, the accuracy of the proposed approach was generally higher than that of K-means.

With real data, we do not know the true number of underlying clusters with 100% confidence. In our application to the FHS samples, we allowed for more clusters and selected $K=13$ considering that there were more than 4000 genes. If there is an approximately reasonable range of possible numbers of clusters available from prior analyses, e.g. at least 10 but no more than 20, we may use that as the candidate values of $K$ in the Silhouette method and the Elbow method. Another lesson that we learned from the FHS application was that we want more regulators in the regression model to capture potentially more factors that have an impact on the clustering structure of GE. Generally speaking, improvement in the imputation quality of the regulation relationship regression model would lead to a more accurate clustering result.

One of the assumptions of the ANCut.overlap clustering approach is that the correlation between GEs comes mostly from the measured regulators. We recommend assessing the imputation quality of GE using the measured regulators based on the overlapping individuals, as shown in Fig. 4 for our application study. Even though the imputation quality measured as ${R}^{2}$ varies a lot across the genes, with a median level of 0.12 and a maximum of larger than 0.80, we performed a global co-expression analysis of all these 2179 genes whose expression levels were predicted by CpG methylation or SNP profiles, regardless of their imputation quality. This is because we have gained confidence from our simulation analysis that even when the imputation quality had a median level as low as 0.05 or below, and the maximum of no higher than 0.40 (Table 9), there is still benefit in using our approach in terms of improving the overall clustering accuracy compared to K-means.

In our application to FHS data, one potential weakness comes from the imputation of the missing DNA methylation data. Some FHS participants are related, inducing correlation between observations. The rationale of MethyLImp lies in the observation that methylation levels show a high degree of inter-sample correlation, and the imputation exploits the inter-sample correlation [24, 25]. It is likely that the family correlation among the FHS data influenced the imputation accuracy of MethyLImp, which further affected the accuracy of the GE-methylation regulation regression and the ANCut.overlap clustering.

The other limitation in our FHS analysis is that we omitted genes that were not predicted by CpG methylation or SNP profiles. For those genes, the predicted values $\widehat{Y}=X\widehat{\beta }$ were just sample means because there was no regulation effect from the measured CpGs or SNPs, according to the elastic net regularization result. The corresponding elements of those genes in the similarity matrix $\widehat{S}$ would be calculated based on their sample means. In this case where the measured regulators are not predictive of the GEs at all, then one may choose to perform clustering analysis based on their observed values only, as what we did in the FHS analysis, without having to incorporate information from the measured regulators. But in this way, we performed GE clustering of only the genes that were predicted by the measured CpGs and SNPs regulators. We might miss some genes that have important biological functions but were not regulated by the measured CpGs and SNPs.

Our proposed approach inevitably has limitations, including the requirement on the overlapping proportion, sample size, and the strength of the GE-regulator relationship. Moreover, GE itself is a cascade, in which the expression of some genes influences the expression of others. Our current approach does not take this into consideration because we model the GE-regulator relationship one gene at a time. However, our approach allows the integration of multi-omic data without restricting to individuals that are common in all omic datasets. This improves GE clustering with the assistance of regulatory data that allows for different but partially overlapping sets of individuals to contribute omics data.

## Conclusion

In this study, we propose a novel approach for clustering GE with the assistance of regulatory data that allowed for different but partially overlapping sets of individuals to be included in different omics data. This is achieved through (1) decomposing GE into the regulated component and the other component that is independent of the measured regulators, where the regulated component is obtained using a GE-regulator transition matrix fit based on the overlapping individuals, and (2) optimizing a clustering goodness-of-fit objective function which incorporates information based on both the regulated component and the non-regulated component. Our simulation study showed that this novel approach yielded competitive clustering accuracy compared to the alternative K-means approach, and the ANCut approach which can only be applied when there was no missingness.

Currently, the approach does not consider the possible regulation relationships between the measured GE regulators. However, if the regulation relationship is strong and is believed to be critical to the clustering structure of GE, we may want to incorporate that relationship. For example, if the regulation relationship between the SNP genotype and CpG methylation plays an essential role in the GE clustering, then we may decompose methylation into the regulated component and the non-regulated component that is independent of the genotype. Because we need to include additional parameters to model this relationship, there is some trade-off between the clustering accuracy and statistical power.

We provide an implementation of our proposed approach ANCut.overlap as an R package available in the GitHub respository https://github.com/WQ-Jiang/ANCut-overlap.

## Supplementary Information

**Additional file 1:** **Table S1.** Imputation quality under various simulation coefficient settings with fixed  *p*=500. **Table S2.** Clustering accuracy under various simulation coefficient settings with fixed *p*=500. **Table S3.** Imputation quality under various simulation coefficient settings with fixed *p*=500 and pcnt = 1/3. **Table S4.** Clustering accuracy under various simulation coefficient settings with fixed *p*=500, K=3 and pcnt =1/3. **Table S5.** Imputation quality under the poor imputation quality scenario with various simulation coefficient settings. **Table S6.** Clustering accuracy under the poor imputation quality scenario with various simulation coefficient settings. **Figure S1. **Distribution of selected genes in FHS samples. **Figure S2.** Histogram of the distance between the methylation sites and their mapped genes. **Figure S3. **Distribution of beta values of methylation sites mapped to selected genes in FHS samples. **Figure S4.** KS test on genes of the lowest GSEA p-value based on assisted clustering.  

## Data Availability

The FHS dataset that supports the findings of this manuscript is available on dbGap (dbGaP Study Accession: phs000007.v32.p13, https://www.ncbi.nlm.nih.gov/projects/gap/cgi-bin/study.cgi?study_id=phs000007.v32.p13).

## Abbreviations

GE: gene expression
ANCut: Assisted Normalized Cut
WCSS: within-cluster sum of squares
SA: simulated annealing
FHS: Framingham Heart Study
CBC: complete blood count
PLS: Partial Least Square
SNP: single nucleotide polymorphism
MAF: minor allele frequency
TPM: transcripts per million RNA molecules
FEV1: volume of air that can forcibly be blown out in first 1 s, after full inspiration
FVC: forced vital capacity
COPD: chronic obstructive pulmonary disease
GSEA: gene set enrichment analysis
TWAS: transcriptome-wide association study
ORA: over-representation analysis

## Declarations

The Framingham Heart Study was approved by the Boston University Medical Campus Institutional Review Board and all participants provided written informed consent. Only adult participants’ data were analyzed in this manuscript.
