# Supplementary material for: Assisted clustering of gene expression data using regulatory data from partially overlapping sets of individuals
Source: BMC Genomics. 2022 Dec 10;23:819. doi: 10.1186/s12864-022-09026-1 (PMC9734806; doi:10.1186/s12864-022-09026-1)
Supplement: Supplementary file 1 — Additional file 1: Table S1. Imputation quality under various simulation coefficient settings with fixed p=500. Table S2. Clustering accuracy under various simulation coefficient settings with fixed p=500. Table S3. Imputation quality under various simulation coefficient settings with fixed p=500 and pcnt = 1/3. Table S4. Clustering accuracy under various simulation coefficient settings with fixed p=500, K=3 and pcnt =1/3. Table S5. Imputation quality under the poor imputation quality scenario with various simulation coefficient settings. Table S6. Clustering accuracy under the poor imputation quality scenario with various simulation coefficient settings. Figure S1. Distribution of selected genes in FHS samples. Figure S2. Histogram of the distance between the methylation sites and their mapped genes. Figure S3. Distribution of beta values of methylation sites mapped to selected genes in FHS samples. Figure S4. KS test on genes of the lowest GSEA p-value based on assisted clustering. [file 12864_2022_9026_MOESM1_ESM.docx]

**RESULTS**

**Simulation studies**

Scenario (I)

Table S1 summarizes the GE imputation quality under different simulation settings of Scenario (I) based on 100 replicates. Correlation coefficient between methylation of CpGs in the same cluster, $\mathrm{corr}$, is set to be 0.1. The proportion of the non-overlapping samples with available GE data $f$ is set to be 0.5. As presented in Table S1, the maximum imputation quality can be as high as 0.7 under certain settings but the median remains as low as 0.1. This is due to how we generate the data – only 5% of the genes can be well imputed, whereas for the others the imputation quality is poor.

Table S1. Imputation quality under various simulation coefficient settings with fixed $p=500$

Table S1(a). Overlap proportion $\mathrm{pcnt}=1/3$

| Median $R^{2}$ (range) | | $q$ | | | | | |
| --- | --- | --- | --- | --- | --- | --- | --- |
|  |  | 200 | 400 | 500 | 600 | 800 | 1000 |
| $n$ | 300 | 0.056  (1e-06, 0.65) | 0.039  (3e-07, 0.53) | 0.036  (1e-07, 0.42) | 0.036  (1e-06, 0.52) | 0.033  (5e-07, 0.56) | 0.039  (4e-07, 0.56) |
|  | 500 | 0.065  (3e-06, 0.66) | 0.059  (2e-06, 0.53) | 0.053  (2e-07, 0.6) | 0.043  (9e-07, 0.52) | 0.044  (3e-06, 0.57) | 0.041  (2e-06, 0.45) |
|  | 1000 | 0.099  (1e-08, 0.72) | 0.073  (4e-09, 0.6) | 0.071  (1e-05, 0.57) | 0.066  (5e-07, 0.6) | 0.069  (3e-06, 0.53) | 0.063  (6e-05, 0.5) |
|  | 1200 | 0.111  (6e-07, 0.75) | 0.083  (1e-07, 0.6) | 0.084  (1e-04, 0.58) | 0.087  (2e-06, 0.63) | 0.076  (7e-10, 0.57) | 0.06  (2e-06, 0.57) |
|  | 1500 | 0.131  (8e-05, 0.78) | 0.1  (2e-05, 0.63) | 0.097  (2e-06, 0.64) | 0.088  (6e-05, 0.55) | 0.074  (3e-06, 0.57) | 0.069  (1e-08, 0.52) |

Table S1(b). Overlap proportion $\mathrm{pcnt}=1/5$

| Median $R^{2}$ (range) | | $q$ | | | | | |
| --- | --- | --- | --- | --- | --- | --- | --- |
|  |  | 200 | 400 | 500 | 600 | 800 | 1000 |
| $n$ | 300 | 0.041  (1e-06, 0.59) | 0.03  (2e-07, 0.47) | 0.031  (2e-08, 0.48) | 0.03  (2e-07, 0.47) | 0.025  (2e-09, 0.49) | 0.027  (6e-07, 0.45) |
|  | 500 | 0.051  (1e-07, 0.65) | 0.042  (2e-05, 0.48) | 0.034  (2e-08, 0.5) | 0.037  (9e-06, 0.48) | 0.034  (6e-08, 0.5) | 0.033  (7e-07, 0.48) |
|  | 1000 | 0.082  (2e-06, 0.67) | 0.06  (2e-07, 0.5) | 0.051  (7e-06, 0.62) | 0.049  (3e-07, 0.5) | 0.046  (3e-07, 0.49) | 0.042  (6e-07, 0.53) |
|  | 1200 | 0.101  (6e-09, 0.67) | 0.064  (3e-06, 0.56) | 0.055  (5e-06, 0.57) | 0.057  (3e-07, 0.49) | 0.055  (1e-07, 0.52) | 0.043  (3e-06, 0.52) |
|  | 1500 | 0.112  (8e-08, 0.7) | 0.08  (1e-06, 0.65) | 0.069  (3e-06, 0.53) | 0.061  (6e-06, 0.54) | 0.055  (8e-07, 0.51) | 0.057  (1e-07, 0.52) |

Table S1(c). Overlap proportion $\mathrm{pcnt}=1/9$

| Median $R^{2}$ (range) | | $q$ | | | | | |
| --- | --- | --- | --- | --- | --- | --- | --- |
|  |  | 200 | 400 | 500 | 600 | 800 | 1000 |
| $n$ | 300 | 0.019  (1e-07, 0.42) | 0.018  (2e-07, 0.43) | 0.023  (6e-07, 0.45) | 0.014  (2e-08, 0.43) | 0.017  (1e-06, 0.48) | 0.015  (2e-06, 0.38) |
|  | 500 | 0.038  (1e-05, 0.57) | 0.029  (4e-07, 0.54) | 0.024  (2e-08, 0.45) | 0.023  (3e-07, 0.47) | 0.021  (7e-07, 0.51) | 0.022  (2e-08, 0.43) |
|  | 1000 | 0.061  (6e-05, 0.56) | 0.043  (7e-06, 0.53) | 0.038  (3e-07, 0.48) | 0.039  (6e-08, 0.51) | 0.035  (1e-08, 0.42) | 0.032  (4e-07, 0.48) |
|  | 1500 | 0.071  (2e-06, 0.68) | 0.048  (4e-07, 0.52) | 0.052  (1e-07, 0.55) | 0.041  (2e-07, 0.51) | 0.04  (3e-07, 0.43) | 0.041  (4e-07, 0.49) |
|  | 3000 | 0.108  (6e-05, 0.66) | 0.08  (2e-08, 0.59) | 0.063  (3e-07, 0.61) | 0.068  (5e-06, 0.52) | 0.061  (4e-07, 0.52) | 0.053  (3e-07, 0.47) |

Summary statistics of clustering accuracy are presented in Table S2. Median values are computed based on 100 replicates.

Table S2. Clustering accuracy under various simulation coefficient settings with fixed $p=500$

| Parameters | | | | Median Accuracy Measure $M_{accuracy}$ | | | | | |
| --- | --- | --- | --- | --- | --- | --- | --- | --- | --- |
| $n$ | $q$ | $K$ | $pcnt$ | ANCut | ANCut.subset | ANCut.overlap | ANCut.silh | ANCut.elbow | K-means |
| 300 | 200 | 3 | 1/3 | 52.0% | 41.7% | 43.5% | 19% (K=2) | 18.7% (K=2) | 23.9% |
| 1500 | 200 | 3 | 1/3 | 69.6% | 61.8% | 63.0% | 63% (K=3) | 62.6% (K=3) | 36.3% |
| 300 | 500 | 3 | 1/3 | 55.0% | 38.4% | 41.1% | 39.9% (K=3) | 41.1% (K=3) | 30.3% |
| 1500 | 500 | 3 | 1/3 | 64.6% | 57.1% | 58.3% | 58.3% (K=3) | 58.6% (K=3) | 25.3% |
| 300 | 1000 | 3 | 1/3 | 62.6% | 42.8% | 44.7% | 43.7% (K=3) | 45.3% (K=3) | 44.1% |
| 1500 | 1000 | 3 | 1/3 | 71.5% | 64.7% | 67.2% | 67.1% (K=3) | 67.6% (K=3) | 28.6% |
| 300 | 200 | 5 | 1/3 | 66.0% | 48.5% | 51.8% | 10.9% (K=2) | 54.6% (K=5) | 45.0% |
| 1500 | 200 | 5 | 1/3 | 73.7% | 70.3% | 72.3% | 72.2% (K=5) | 70.5% (K=6) | 31.3% |
| 300 | 500 | 5 | 1/3 | 61.5% | 51.7% | 52.6% | 46% (K=4) | 33.6% (K=3) | 46.9% |
| 1500 | 500 | 5 | 1/3 | 67.3% | 63.3% | 67.9% | 66.9% (K=5) | 67.7% (K=5) | 22.6% |
| 300 | 1000 | 5 | 1/3 | 70.2% | 51.2% | 55.0% | 10.9% (K=2) | 10.5% (K=2) | 53.6% |
| 1500 | 1000 | 5 | 1/3 | 76.1% | 68.8% | 74.9% | 73.7% (K=5) | 71.4% (K=5) | 38.4% |
|  |  |  |  |  |  |  |  |  |  |
| 300 | 200 | 3 | 1/5 | 58.9% | 33.9% | 35.5% | 35.7% (K=3) | 35.8% (K=3) | 37.8% |
| 1500 | 200 | 3 | 1/5 | 69.7% | 58.3% | 57.8% | 58.2% (K=3) | 57.9% (K=3) | 32.6% |
| 300 | 500 | 3 | 1/5 | 59.0% | 28.4% | 30.9% | 17.4% (K=2) | 16.8% (K=2) | 34.6% |
| 1500 | 500 | 3 | 1/5 | 66.7% | 56.3% | 58.3% | 58.3% (K=3) | 58.3% (K=3) | 24.4% |
| 300 | 1000 | 3 | 1/5 | 60.1% | 39.6% | 45.2% | 44.5% (K=3) | 44.5% (K=3) | 45.0% |
| 1500 | 1000 | 3 | 1/5 | 64.9% | 57.0% | 57.0% | 56.6% (K=3) | 56.6% (K=3) | 38.4% |
| 300 | 200 | 5 | 1/5 | 68.2% | 44.4% | 46.4% | 8.5% (K=2) | 7.4% (K=2) | 38.8% |
| 1500 | 200 | 5 | 1/5 | 76.2% | 64.7% | 69.8% | 71.7% (K=6) | 69.7% (K=5) | 33.4% |
| 300 | 500 | 5 | 1/5 | 67.4% | 43.9% | 45.8% | 7% (K=2) | 28.6% (K=3) | 38.2% |
| 1500 | 500 | 5 | 1/5 | 70.0% | 58.7% | 64.4% | 59% (K=5) | 65.9% (K=6) | 33.3% |
| 300 | 1000 | 5 | 1/5 | 67.1% | 41.2% | 47.6% | 6.2% (K=2) | 6.7% (K=2) | 35.6% |
| 1500 | 1000 | 5 | 1/5 | 75.2% | 60.1% | 66.6% | 66.8% (K=5) | 66.9% (K=5) | 23.7% |
|  |  |  |  |  |  |  |  |  |  |
| 300 | 200 | 3 | 1/9 | 57.9% | 16.6% | 21.9% | 9.7% (K=2) | 9.2% (K=2) | 18.9% |
| 1500 | 200 | 3 | 1/9 | 72.2% | 54.3% | 59.3% | 59% (K=3) | 60.1% (K=3) | 36.8% |
| 300 | 500 | 3 | 1/9 | 60.9% | 20.4% | 26.3% | 22.7% (K=3) | 25% (K=3) | 45.1% |
| 1500 | 500 | 3 | 1/9 | 65.9% | 49.6% | 53.2% | 53.2% (K=3) | 53.3% (K=3) | 34.1% |
| 300 | 1000 | 3 | 1/9 | 60.9% | 19.5% | 25.5% | 7.9% (K=2) | 8.6% (K=2) | 29.8% |
| 1500 | 1000 | 3 | 1/9 | 61.4% | 41.6% | 44.1% | 45.4% (K=3) | 41.2% (K=3) | 14.7% |
| 300 | 200 | 5 | 1/9 | 68.8% | 40.4% | 45.1% | 6.3% (K=2) | 30.9% (K=3) | 36.6% |
| 1500 | 200 | 5 | 1/9 | 74.5% | 63.1% | 64.7% | 64.2% (K=5) | 65.1% (K=5) | 24.2% |
| 300 | 500 | 5 | 1/9 | 67.2% | 40.6% | 43.1% | 6.8% (K=2) | 7.2% (K=2) | 33.6% |
| 1500 | 500 | 5 | 1/9 | 72.9% | 57.7% | 61.5% | 63.4% (K=6) | 14.6% (K=2) | 31.2% |
| 300 | 1000 | 5 | 1/9 | 66.9% | 37.7% | 40.9% | 4% (K=2) | 5.2% (K=2) | 40.8% |
| 1500 | 1000 | 5 | 1/9 | 76.9% | 55.9% | 55.4% | 13.2% (K=2) | 12.2% (K=2) | 25.5% |

Column notations:

*ANCut* uses Hidalgo’s assisted clustering approach to cluster gene expression, assuming there is no missing data. This serves as the “gold standard” as we compare various clustering approaches because this approach uses the largest amount of data – the entire gene expression data matrix and methylation data matrix in Figure 1 of the main paper. The true number of clusters $K$ is assumed to be known.

*ANCut.subset* also uses Hidalgo’s assisted clustering approach to cluster gene expression but uses only the overlapping samples that have both GE and methylation data.

*ANCut.overlap* uses the proposed approach assuming only a subset of the data is available, i.e. the $X$ and $Y$ matrices in Figure 1 of the main paper. With the $\left( pcnt*n \right)$ overlapping individuals, we can construct a regression model between GE ($Y^{O}$) and the methylation regulators ($X^{O}$) to improve GE clustering. The true number of clusters $K$ is assumed to be known.

*ANCut.silh* uses the proposed approach (ANCut.overlap) with the Silhouette method to select the optimal number of clusters.

*ANCut.elbow* uses the proposed approach (ANCut.overlap) with the Elbow method to select the optimal number of clusters.

*K-means* uses K-means method to cluster GE, using only the $Y$ matrix (with missing data) in Figure 1 of the main paper. The true number of clusters $K$ is assumed to be known.

To further evaluate the simulation, we consider varying the correlation coefficient between methylation of CpGs $\mathrm{corr}$, and varying the proportion of the non-overlapping samples with available GE data $f$. Table S3 summarizes the GE imputation quality under additional simulation settings of Scenario (I) with varying $\mathrm{corr}$ and $f$. Results are based on 100 replicates.

Table S3. Imputation quality under various simulation coefficient settings with fixed $p=500$ and $\mathrm{pcnt}=1/3$

| Median $R^{2}$ (range) | | $q$ | | |
| --- | --- | --- | --- | --- |
|  |  | $200$ | $500$ | $1000$ |
| $\mathrm{corr}=0.3, f=0.5$ | $n=300$ | 0.079  (3e-07 – 0.81) | 0.077  (3e-07 – 0.82) | 0.109  (5e-07 – 0.81) |
|  | $n=1500$ | 0.156  (2e-06 – 0.87) | 0.136  (6e-05 – 0.81) | 0.134  (7e-06 – 0.78) |
| $\mathrm{corr}=0.1, f=0.3$ | $n=300$ | 0.049  (4e-07 – 0.58) | 0.036  (1e-07 – 0.57) | 0.032  (7e-07 – 0.46) |
|  | $n=1500$ | 0.152  (6e-06 – 0.76) | 0.092  (2e-08 – 0.58) | 0.061  (9e-05 – 0.56) |
| $\mathrm{corr}=0.1, f=0.7$ | $n=300$ | 0.053  (2e-07 – 0.59) | 0.045  (1e-07 – 0.56) | 0.029  (1e-07 – 0.53) |
|  | $n=1500$ | 0.143  (1e-06 – 0.78) | 0.096  (1e-05 – 0.65) | 0.073  (4e-07 – 0.53) |

Summary statistics of clustering accuracy are presented in Table S4. Median values are computed based on 100 replicates.

Table S4. Clustering accuracy under various simulation coefficient settings with fixed $p=500, K=3$and$\mathrm{pcnt}=1/3$

| Parameters | | | | Median Accuracy Measure $M_{accuracy}$ | | | | | |
| --- | --- | --- | --- | --- | --- | --- | --- | --- | --- |
| $corr$ | $f$ | $n$ | $q$ | ANCut | ANCut.subset | ANCut.overlap | ANCut.silh | ANCut.elbow | K-means |
| 0.3 | 0.5 | 300 | 200 | 58.2% | 43.6% | 45.9% | 45.6% (K=3) | 46.3% (K=3) | 37.4% |
|  |  | 1500 | 200 | 63.6% | 59.7% | 60.6% | 60.3% (K=4) | 59.8% (K=3) | 30.7% |
|  |  | 300 | 500 | 60.9% | 45.3% | 47.2% | 48.2% (K=3) | 46.5% (K=3) | 29.1% |
|  |  | 1500 | 500 | 66.7% | 60.2% | 59.7% | 59.9% (K=3) | 59.4% (K=3) | 31.6% |
|  |  | 300 | 1000 | 54.9% | 32.0% | 32.4% | 32.0% (K=3) | 32.1% (K=3) | 29.2% |
|  |  | 1500 | 1000 | 61.0% | 56.6% | 57.8% | 57.2% (K=3) | 58.1% (K=3) | 20.0% |
| 0.1 | 0.3 | 300 | 200 | 66.0% | 59.8% | 59.1% | 58.5% (K=3) | 59.3% (K=3) | 40.9% |
|  |  | 1500 | 200 | 65.8% | 66.5% | 65.7% | 65.8% (K=3) | 65.7% (K=3) | 43.1% |
|  |  | 300 | 500 | 67.6% | 57.0% | 57.9% | 57.7% (K=3) | 57.7% (K=3) | 32.8% |
|  |  | 1500 | 500 | 68.4% | 67.6% | 66.4% | 66.4% (K=3) | 66.4% (K=3) | 40.5% |
|  |  | 300 | 1000 | 65.5% | 57.4% | 59.1% | 59.4% (K=3) | 59.0% (K=3) | 30.2% |
|  |  | 1500 | 1000 | 63.0% | 60.3% | 65.0% | 65.5% (K=3) | 61.7% (K=3) | 33.9% |
| 0.1 | 0.7 | 300 | 200 | 57.7% | 42.5% | 43.8% | 36.0% (K=2) | 44.3% (K=3) | 26.8% |
|  |  | 1500 | 200 | 67.9% | 64.2% | 63.8% | 64.1% (K=3) | 64.6% (K=3) | 40.6% |
|  |  | 300 | 500 | 60.9% | 46.6% | 48.4% | 49.8% (K=3) | 49.4% (K=3) | 35.5% |
|  |  | 1500 | 500 | 72.8% | 66.4% | 66.4% | 66.5% (K=3) | 66.1% (K=3) | 39.7% |
|  |  | 300 | 1000 | 55.1% | 39.3% | 41.8% | 42.1% (K=3) | 22.1% (K=2) | 36.2% |
|  |  | 1500 | 1000 | 57.8% | 53.6% | 54.7% | 56.4% (K=4) | 55.1% (K=3) | 20.2% |

Please refer to Table S2 for explanation of the column notations.

Scenario (II)

Table S5 summarizes the imputation quality with various parameter settings under the poor imputation quality scenario based on 100 replicates. The number of genes $p=500$ remains fixed.

Table S5. Imputation quality under the poor imputation quality scenario with various simulation coefficient settings

Table S5(a). Overlap proportion $\mathrm{pcnt}=1/3$

| Median $R^{2}$ (range) | | $q$ | | | | | |
| --- | --- | --- | --- | --- | --- | --- | --- |
|  |  | 200 | 400 | 500 | 600 | 800 | 1000 |
| $n$ | 300 | 0.011  (3e-07, 0.21) | 0.013  (6e-08, 0.20) | 0.009  (6e-09, 0.21) | 0.008  (3e-07, 0.18) | 0.008  (2e-07, 0.18) | 0.009  (4e-09, 0.21) |
|  | 500 | 0.013  (6e-11, 0.25) | 0.011  (3e-07, 0.19) | 0.007  (1e-09, 0.22) | 0.009  (4e-08, 0.20) | 0.010  (5e-07, 0.29) | 0.007  (9e-08, 0.16) |
|  | 1000 | 0.016  (2e-09, 0.33) | 0.009  (1e-08, 0.25) | 0.013  (5e-07, 0.25) | 0.009  (1e-07, 0.27) | 0.011  (2e-07, 0.21) | 0.007  (3e-09, 0.15) |
|  | 1200 | 0.022  (5e-07, 0.28) | 0.014  (7e-08, 0.22) | 0.014  (2e-07, 0.21) | 0.011  (3e-10, 0.19) | 0.011  (4e-08, 0.20) | 0.012  (3e-08, 0.23) |
|  | 1500 | 0.023  (4e-07, 0.31) | 0.019  (2e-07, 0.31) | 0.014  (2e-07, 0.25) | 0.011  (3e-08, 0.29) | 0.012  (2e-07, 0.25) | 0.008  (4e-07, 0.23) |

Table S5(b). Overlap proportion $\mathrm{pcnt}=1/5$

| Median $R^{2}$ (range) | | $q$ | | | | | |
| --- | --- | --- | --- | --- | --- | --- | --- |
|  |  | 200 | 400 | 500 | 600 | 800 | 1000 |
| $n$ | 300 | 0.007  (2e-07, 0.2) | 0.005  (5e-08, 0.19) | 0.005  (6e-09, 0.17) | 0.006  (4e-08, 0.27) | 0.006  (6e-09, 0.17) | 0.006  (5e-08, 0.17) |
|  | 500 | 0.008  (2e-08, 0.22) | 0.007  (5e-08, 0.21) | 0.006  (7e-08, 0.21) | 0.008  (3e-09, 0.13) | 0.004  (7e-08, 0.15) | 0.006  (7e-08, 0.15) |
|  | 1000 | 0.013  (7e-08, 0.26) | 0.01  (4e-09, 0.16) | 0.009  (3e-10, 0.19) | 0.007  (5e-07, 0.2) | 0.007  (2e-07, 0.14) | 0.008  (6e-09, 0.17) |
|  | 1200 | 0.012  (2e-08, 0.23) | 0.009  (4e-07, 0.19) | 0.009  (2e-07, 0.16) | 0.01  (2e-07, 0.16) | 0.006  (1e-11, 0.14) | 0.005  (6e-09, 0.17) |
|  | 1500 | 0.017  (9e-07, 0.31) | 0.011  (1e-08, 0.23) | 0.009  (5e-08, 0.22) | 0.01  (2e-07, 0.21) | 0.008  (2e-08, 0.2) | 0.007  (4e-07, 0.14) |

Table S5(c). Overlap proportion $\mathrm{pcnt}=1/9$

| Median $R^{2}$ (range) | | $q$ | | | | | |
| --- | --- | --- | --- | --- | --- | --- | --- |
|  |  | 200 | 400 | 500 | 600 | 800 | 1000 |
| $n$ | 300 | 0.006  (2e-09, 0.17) | 0.004  (1e-08, 0.1) | 0.005  (8e-07, 0.15) | 0.004  (9e-07, 0.09) | 0.004  (3e-07, 0.09) | 0.005  (3e-09, 0.14) |
|  | 500 | 0.005  (3e-07, 0.19) | 0.004  (2e-08, 0.15) | 0.004  (4e-09, 0.15) | 0.004  (2e-07, 0.11) | 0.004  (2e-08, 0.11) | 0.003  (2e-08, 0.13) |
|  | 1000 | 0.01  (1e-07, 0.21) | 0.005  (4e-08, 0.19) | 0.006  (6e-08, 0.19) | 0.004  (3e-08, 0.14) | 0.005  (7e-07, 0.16) | 0.005  (2e-07, 0.17) |
|  | 1500 | 0.008  (3e-08, 0.17) | 0.006  (1e-07, 0.19) | 0.008  (5e-10, 0.18) | 0.005  (2e-07, 0.14) | 0.004  (3e-08, 0.13) | 0.005  (3e-08, 0.12) |
|  | 3000 | 0.014  (2e-07, 0.27) | 0.009  (7e-07, 0.32) | 0.011  (2e-07, 0.21) | 0.01  (3e-07, 0.21) | 0.007  (8e-07, 0.19) | 0.007  (5e-07, 0.16) |

Comparison of clustering accuracy between different clustering approaches is presented in Table S6. Median values are computed based on 100 replicates. The number of genes $p=500$, the correlation coefficient between methylation of CpGs $corr=0.1$, and varying the proportion of the non-overlapping samples with available GE data $f=0.5$ remain fixed.

Table S6. Clustering accuracy under the poor imputation quality scenario with various simulation coefficient settings

| Parameters | | | | Median Accuracy Measure $M_{accuracy}$ | | | | | |
| --- | --- | --- | --- | --- | --- | --- | --- | --- | --- |
| $n$ | $q$ | $K$ | $pcnt$ | ANCut | ANCut.subset | ANCut.overlap | ANCut.silh | ANCut.elbow | K-means |
| 500 | 200 | 3 | 1/3 | 43.1% | 23.9% | 22.4% | 8.1% (K=2) | 8.1% (K=2) | 11.5% |
| 1500 | 200 | 3 | 1/3 | 47.6% | 40.1% | 40.2% | 40.2% (K=3) | 40.9% (K=3) | 11.4% |
| 3000 | 200 | 3 | 1/3 | 62.9% | 54.8% | 54.1% | 54.4% (K=3) | 54.1% (K=3) | 12.1% |
| 500 | 500 | 3 | 1/3 | 41.0% | 24.1% | 23.1% | 8.6% (K=2) | 8.7% (K=2) | 11.6% |
| 1500 | 500 | 3 | 1/3 | 49.8% | 37.4% | 38.1% | 37.9% (K=3) | 37.9% (K=3) | 11.4% |
| 3000 | 500 | 3 | 1/3 | 52.4% | 50.4% | 50.9% | 50.5% (K=3) | 51.4% (K=4) | 11.4% |
| 500 | 1000 | 3 | 1/3 | 42.7% | 16.2% | 20.2% | 4.5% (K=2) | 5.6% (K=2) | 11.3% |
| 1500 | 1000 | 3 | 1/3 | 52.3% | 44.6% | 46.2% | 46.2% (K=3) | 45.9% (K=3) | 11.5% |
| 3000 | 1000 | 3 | 1/3 | 57.0% | 47.2% | 46.5% | 52% (K=4) | 47% (K=3) | 11.6% |
| 500 | 200 | 5 | 1/3 | 48.8% | 40.2% | 40.2% | 4.1% (K=2) | 2.7% (K=2) | 36.3% |
| 1500 | 200 | 5 | 1/3 | 63.3% | 54.2% | 53.1% | 53.5% (K=5) | 6.1% (K=2) | 36.3% |
| 3000 | 200 | 5 | 1/3 | 63.1% | 63.7% | 64.7% | 63.4% (K=5) | 62.7% (K=6) | 36.5% |
| 500 | 500 | 5 | 1/3 | 51.7% | 40.2% | 40.8% | 3.8% (K=2) | 4.5% (K=2) | 36.4% |
| 1500 | 500 | 5 | 1/3 | 58.9% | 52.3% | 54.7% | 44.4% (K=4) | 48.8% (K=5) | 36.2% |
| 3000 | 500 | 5 | 1/3 | 65.1% | 57.2% | 58.7% | 58.1% (K=5) | 58.4% (K=5) | 36.4% |
| 500 | 1000 | 5 | 1/3 | 49.6% | 39.1% | 38.9% | 2.2% (K=2) | 1.8% (K=2) | 36.3% |
| 1500 | 1000 | 5 | 1/3 | 62.6% | 51.3% | 53.9% | 44.7% (K=4) | 52.8% (K=5) | 36.4% |
| 3000 | 1000 | 5 | 1/3 | 68.1% | 61.4% | 60.6% | 60.2% (K=5) | 59.5% (K=5) | 36.3% |
|  |  |  |  |  |  |  |  |  |  |
| 500 | 200 | 3 | 1/5 | 42.3% | 18.2% | 17.2% | 5.9% (K=2) | 5.9% (K=2) | 11.4% |
| 1500 | 200 | 3 | 1/5 | 56.3% | 37.5% | 40.4% | 41% (K=3) | 39.4% (K=3) | 11.7% |
| 3000 | 200 | 3 | 1/5 | 50.4% | 40.9% | 41.1% | 46.2% (K=4) | 46.9% (K=4) | 11.8% |
| 500 | 500 | 3 | 1/5 | 39.3% | 16.4% | 17.3% | 1.5% (K=2) | 1.2% (K=2) | 11.3% |
| 1500 | 500 | 3 | 1/5 | 47.6% | 33.0% | 33.1% | 29.1% (K=3) | 32.4% (K=3) | 11.4% |
| 3000 | 500 | 3 | 1/5 | 52.9% | 43.7% | 43.4% | 46.8% (K=4) | 43.4% (K=3) | 11.2% |
| 500 | 1000 | 3 | 1/5 | 31.6% | 14.4% | 14.9% | 4.4% (K=2) | 4.4% (K=2) | 11.2% |
| 1500 | 1000 | 3 | 1/5 | 44.2% | 28.0% | 29.4% | 30.5% (K=3) | 9% (K=2) | 11.4% |
| 3000 | 1000 | 3 | 1/5 | 54.7% | 43.3% | 43.9% | 43.6% (K=3) | 43.6% (K=3) | 11.5% |
| 500 | 200 | 5 | 1/5 | 51.0% | 39.3% | 39.9% | 3.7% (K=2) | 24.5% (K=3) | 36.5% |
| 1500 | 200 | 5 | 1/5 | 66.4% | 46.5% | 50.1% | 54.2% (K=5) | 7.8% (K=2) | 36.4% |
| 3000 | 200 | 5 | 1/5 | 65.9% | 58.5% | 59.2% | 60.6% (K=5) | 58.9% (K=5) | 36.5% |
| 500 | 500 | 5 | 1/5 | 57.2% | 38.6% | 38.8% | 2.1% (K=2) | 2.3% (K=2) | 36.4% |
| 1500 | 500 | 5 | 1/5 | 62.0% | 45.2% | 45.8% | 7.1% (K=2) | 6.3% (K=2) | 36.5% |
| 3000 | 500 | 5 | 1/5 | 62.1% | 49.8% | 54.6% | 48.3% (K=5) | 54.7% (K=6) | 36.5% |
| 500 | 1000 | 5 | 1/5 | 55.0% | 38.8% | 38.7% | 3.3% (K=2) | 2.9% (K=2) | 36.6% |
| 1500 | 1000 | 5 | 1/5 | 57.6% | 43.9% | 43.2% | 6.4% (K=2) | 6.5% (K=2) | 36.5% |
| 3000 | 1000 | 5 | 1/5 | 60.2% | 53.0% | 52.5% | 54.6% (K=5) | 52.7% (K=5) | 36.3% |
|  |  |  |  |  |  |  |  |  |  |
| 500 | 200 | 3 | 1/9 | 38.6% | 14.1% | 11.7% | 0.1% (K=2) | 0.2% (K=2) | 11.4% |
| 1500 | 200 | 3 | 1/9 | 46.9% | 26.9% | 27.7% | 6.6% (K=2) | 28.3% (K=3) | 11.6% |
| 3000 | 200 | 3 | 1/9 | 52.9% | 34.1% | 36.7% | 38.6% (K=3) | 35% (K=3) | 11.5% |
| 500 | 500 | 3 | 1/9 | 46.2% | 14.7% | 14.5% | 4.8% (K=2) | 19.5% (K=3) | 12.1% |
| 1500 | 500 | 3 | 1/9 | 47.9% | 17.0% | 22.7% | 6.2% (K=2) | 5.8% (K=2) | 11.5% |
| 3000 | 500 | 3 | 1/9 | 51.1% | 31.3% | 33.9% | 35.4% (K=3) | 38.2% (K=4) | 11.5% |
| 500 | 1000 | 3 | 1/9 | 32.3% | 11.4% | 12.4% | 1% (K=2) | 1.5% (K=2) | 11.6% |
| 1500 | 1000 | 3 | 1/9 | 49.6% | 19.2% | 24.2% | 7.6% (K=2) | 7.7% (K=2) | 11.5% |
| 3000 | 1000 | 3 | 1/9 | 59.8% | 34.7% | 34.9% | 35.1% (K=3) | 35.1% (K=3) | 11.9% |
| 500 | 200 | 5 | 1/9 | 47.9% | 37.8% | 37.7% | 2% (K=2) | 1.1% (K=2) | 36.3% |
| 1500 | 200 | 5 | 1/9 | 61.3% | 39.8% | 40.7% | 4.4% (K=2) | 24.8% (K=3) | 36.3% |
| 3000 | 200 | 5 | 1/9 | 61.8% | 47.6% | 46.8% | 6.6% (K=2) | 7.4% (K=2) | 36.4% |
| 500 | 500 | 5 | 1/9 | 49.6% | 38.0% | 38.5% | 2.5% (K=2) | 2.4% (K=2) | 36.3% |
| 1500 | 500 | 5 | 1/9 | 64.3% | 39.4% | 40.2% | 2.9% (K=2) | 3% (K=2) | 36.4% |
| 3000 | 500 | 5 | 1/9 | 63.3% | 45.3% | 46.7% | 5.4% (K=2) | 5.6% (K=2) | 36.4% |
| 500 | 1000 | 5 | 1/9 | 50.5% | 37.2% | 29.2% | 0.1% (K=2) | 0.7% (K=2) | 36.2% |
| 1500 | 1000 | 5 | 1/9 | 65.2% | 39.0% | 40.1% | 2.5% (K=2) | 4.6% (K=2) | 36.5% |
| 3000 | 1000 | 5 | 1/9 | 61.2% | 43.2% | 44.5% | 26.3% (K=3) | 2.4% (K=2) | 36.4% |

Please refer to Table S2 for explanation of the column notations.

**Application to FHS**

Figure S1 shows the distribution of these 4152 genes in the 5626 FHS individuals

In the scatterplot, the x-axis shows the mean of the GE level averaged over the 5626 FHS participants; the y-axis shows the coefficient of variation of the GE level across FHS participants.


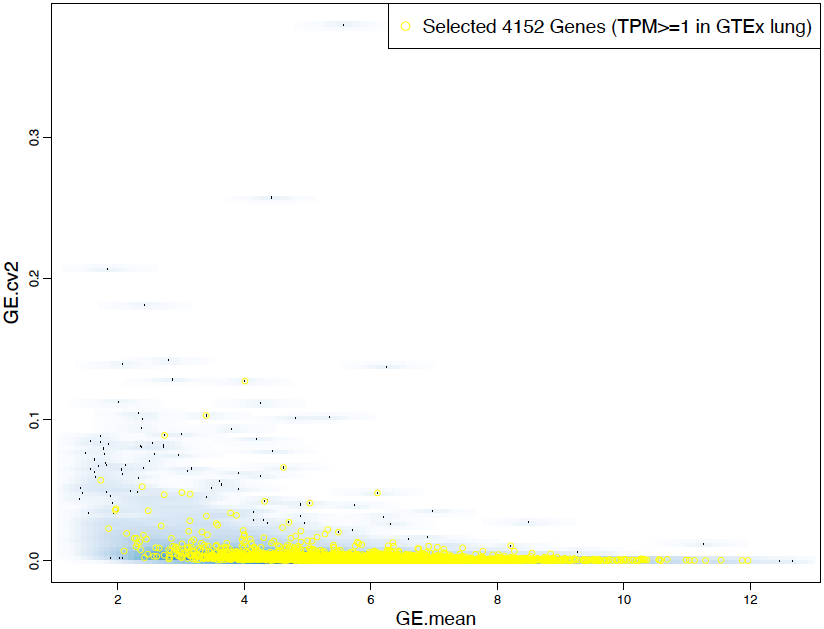


Figure S1. Distribution of selected genes in FHS samples

The blue scatterplot shows the GE distribution of all the 17873 genes measured in FHS;

the yellow circles represent the 4152 selected genes.

Figure S2 shows the distribution of the distance, in base pair, between the methylation sites to their mapped genes is available in the supplementary materials. Figure S3 shows the distribution of beta values of the methylation sites in the 4161 FHS participants can also be found in the supplementary materials. In the scatterplot, the x-axis shows the mean of the beta values averaged over FHS participants; the y-axis shows the coefficient of variation of beta values across FHS samples.


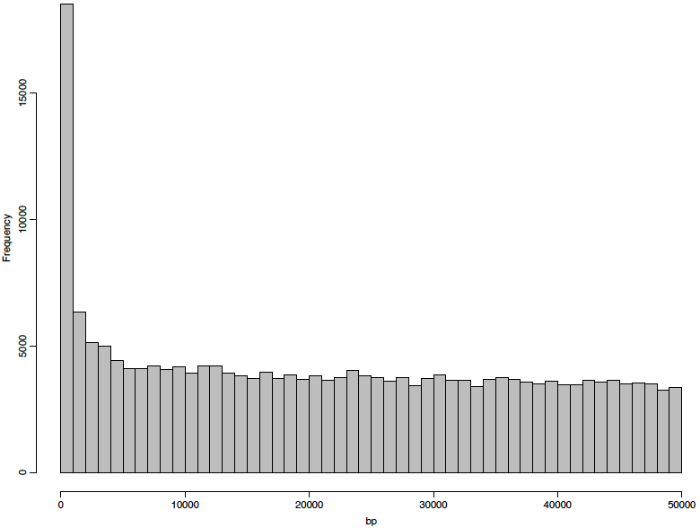


Figure S2. Histogram of the distance between the methylation sites and their mapped genes


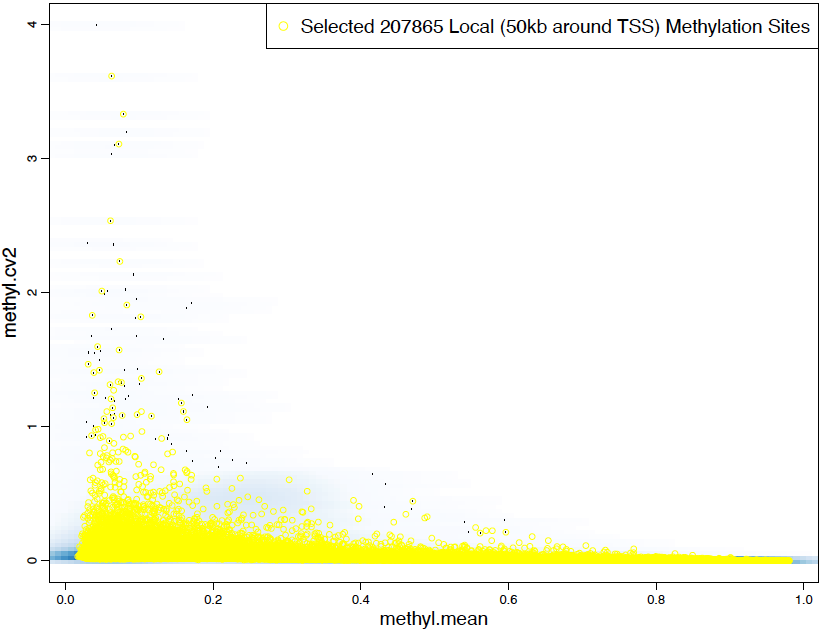


Figure S3. Distribution of beta values of methylation sites mapped to selected genes in FHS samples

The blue scatterplot shows the methylation distribution of all the CpG sites measured in FHS;

the yellow circles represent the 207,865 CpG sites mapped to the 4152 selected genes.

Kolmogorov-Smirnov (KS) test is performed on clusters 11 and 7 from assisted clustering. The KS p values of cluster 11 and 7 are 0.013 and 0.090 respectively. As shown in Figure S4, results confirm the enrichment of these two clusters.


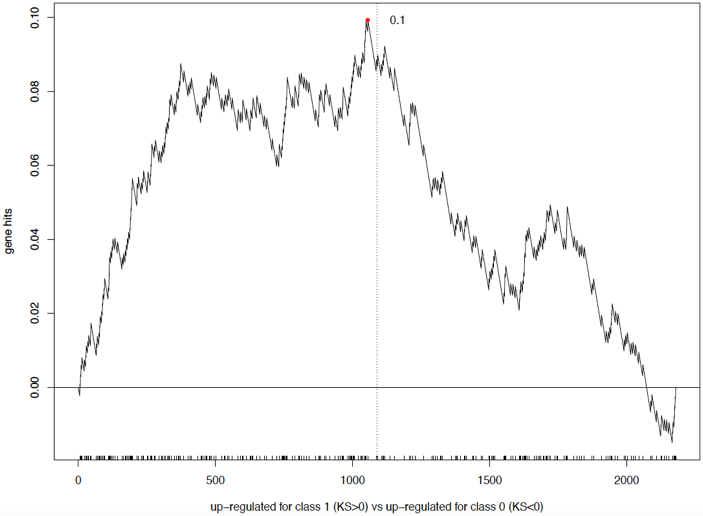

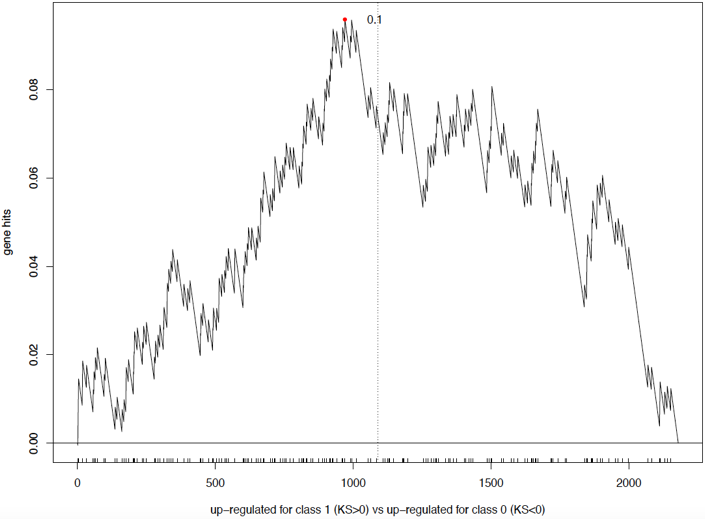


(a) KS test of cluster 11 (b) KS test of cluster 7

Figure S4. KS test on genes of the lowest GSEA p-value based on assisted clustering
